# Assessing subclinical psychopathological and personality traits in a small-scale subsistence society

**DOI:** 10.1093/emph/eoaf033

**Published:** 2025-11-20

**Authors:** Camila Scaff, Charlotte Van Den Driessche, Agustina Bani Cuata, Alberto Vie Tayo, Adrian V Jaeggi

**Affiliations:** Human Ecology Group, Institute of Evolutionary Medicine, University of Zurich, Zurich, Zurich, Switzerland; Laboratoire de Sciences Cognitives et de Psycholinguistique, Département d’études cognitives, ENS, EHESS, CNRS, PSL University, Paris, Ile-de-France, France; University of Cambridge, Center for Human Evolutionary Studies, McDonald Institute for Archaeological Research, Cambridge, Cambridgeshire, UK; Yucumo, Beni Province, Bolivia; Yucumo, Beni Province, Bolivia; Human Ecology Group, Institute of Evolutionary Medicine, University of Zurich, Zurich, Zurich, Switzerland

**Keywords:** cross-cultural psychology, psychopathology, neurodiversity, mental health assessment, evolutionary psychiatry, small scale society

## Abstract

**Background and objectives:**

Are psychiatric conditions linked to Western, Educated, Industrialized, Rich, and Democratic (WEIRD) lifestyles, akin to other “diseases of civilization”, or have they always been part of human variation? Are psychiatric traits always harmful and selected against, or can they be neutral or adaptive in some contexts? Addressing such core questions in evolutionary psychiatry requires examining and quantifying psychiatric symptoms and their subclinical manifestation in radically different cultural and ecological settings, such as small-scale subsistence societies. Available tools designed for the global North are often ill-suited for these communities, failing to translate and to reflect culturally-specific experiences. Here, we present a multi-stage approach for assessing subclinical psychopathological and personality traits among the Tsimaneʼ, an Indigenous forager-horticulturalist population in lowland Bolivia.

**Methodology:**

Building on established questionnaires, we reviewed over 400 items through extensive collaboration with local research assistants, focus groups, and cognitive interviews. We grounded each item in culturally relevant examples and translated them into Tsimaneʼ, ensuring both conceptual accuracy and comprehensibility.

**Results:**

The final instrument consists of 117 items associated in Global North settings with autism spectrum disorder, attention deficit hyperactivity disorder, obsessive-compulsive disorder, schizotypy, depression, anxiety, trauma, substance use, and personality.

**Conclusions and implications:**

This study provides a model for developing culturally sensitive tools to measure mental health traits in small-scale societies. It contributes to evolutionary psychiatry by laying the groundwork for quantifying subclinical psychopathology and personality traits, enabling rigorous tests of evolutionary hypotheses.

## INTRODUCTION

Cross-cultural studies are essential to reveal how psychopathological traits manifest in different contexts. While many mental conditions exhibit a degree of universality, there is notable variation in symptomatology and prognosis [1]. In addition, local understandings of mental health and psychiatric disorders vary significantly across cultures [2]. Here, we present a guide and subsequent survey measuring psychopathological and personality traits in a small-scale subsistence society.

### Theoretical motivation

Evolutionary perspectives are starting to change our understanding of mental health and challenge conventional biomedical narratives [3–6]. One major insight from this perspective is that our physiology and behaviors have adapted to environments quite different from contemporary industrialized societies, and such “mismatch” can dysregulate otherwise normal processes. For instance, low mood is arguably an adaptive response to infection, allowing an organism to conserve energy and direct it toward immune function [7, 8]. However, according to this hypothesis, when inflammation becomes chronic due to obesity, smoking, physical inactivity, and other pro-inflammatory lifestyle factors, the chronic activation of this response could lead to depression [9]; for a more nuanced discussion of the proposed link between inflammation and depression, see [3, 10].

Another major insight is that many long-lasting psychiatric conditions, such as autism spectrum disorder (ASD), schizophrenia, attention deficit hyperactivity disorder (ADHD), and obsessive-compulsive disorder (OCD), may not represent categorical “disorders” but rather the extremes of a spectrum of subclinical traits present in the general population [11]. This forces us to consider what evolutionary forces shaped variation in these traits and whether some that may appear unfavorable in some settings could have been neutral or beneficial in others [3, 12]. Testing such evolutionary hypotheses requires quantitative data on psychopathological traits and their associated subclinical spectra in diverse societies.

### Limitations of currently available surveys

Psychopathology remains a largely subjective field, relying primarily on clinical diagnosis. Mental health conditions are typically evaluated using standardized tools like quantitative questionnaires and semi-structured interviews.

There are significant problems regarding the cross-cultural validity of instruments used to assess psychopathology. First, many instruments treat complex conditions as single constructs via the use of sum-score instruments. For example, the Beck Depression Inventory (BDI) or Hamilton Rating Scale for Depression (HAM-D) use a list of symptoms, with each symptom given a score that is summed to produce a single overall measure of depression severity [13, 14]. However, such approaches oversimplify complex disorders by assuming that all symptoms have equal weight and manifest similarly across contexts. Moreover, most psychopathological conditions are heterogeneous syndromes with high comorbidity [15]. Total survey scores, aiming to reach diagnostic levels quantitatively, dissolve our ability to distinguish individual symptoms and their relation to the local socio-ecology and selective pressures.

Second, most instruments available for screening and assessing psychopathology and mental health were created by and for the Global North [16, 17]. These tools are typically framed in ways that reflect Western cultural norms and experiences, which can lead to misunderstandings or inaccuracies in contexts outside the Global North [18, 19]. For example, questions about workplace stress or urban living environments may not be relevant to individuals living in foraging or subsistence-based communities. Furthermore, some concepts central to Western psychopathology (such as anxiety or stress) may lack direct equivalents in other languages or be expressed differently [2, 20]. This issue is particularly pronounced in small-scale societies, where lifestyle, literacy levels, social organization, and day-to-day activities differ significantly from the expectations, situations, and examples used in Global North instruments, making the direct application of screening tools problematic. Evidence suggests the expression of psychopathological traits is dependent on the local socio-ecology and unique language and expressions (idioms of distress) used by a given population and at a given time [2, 20]. Without acknowledging these culturally specific expressions, there is a risk of pathologizing normal behaviors or missing signs of disorders and distress altogether [21]. Addressing this issue requires developing tools that are culturally sensitive, grounded in local socio-ecologies, and capable of capturing local idioms of distress.

### Goals of the present work

Following previous work on depressive symptoms [8, 22, 23] and personality traits [24, 25], we created a Tsimaneʼ-specific scale to measure subclinical traits associated with common psychiatric conditions. Specifically, we focused on ASD, schizotypal personality, OCD, ADHD, depression, anxiety, trauma, and other behavioral problems such as substance abuse and sleep issues, as well as general personality traits. The primary goal, at this stage of the present study, was to carry out the essential preliminary step of cultural and linguistic adaptation. In other words, our focus was to assess whether constructs and items drawn from standard inventories are comprehensible and meaningful within the Tsimaneʼ cultural and linguistic framework. In the following, we detail the methodology and survey creation process to aid researchers and practitioners interested in collecting quantitative data for studying mental health and psychopathology in diverse settings.

## METHODS

**Figure 1:**
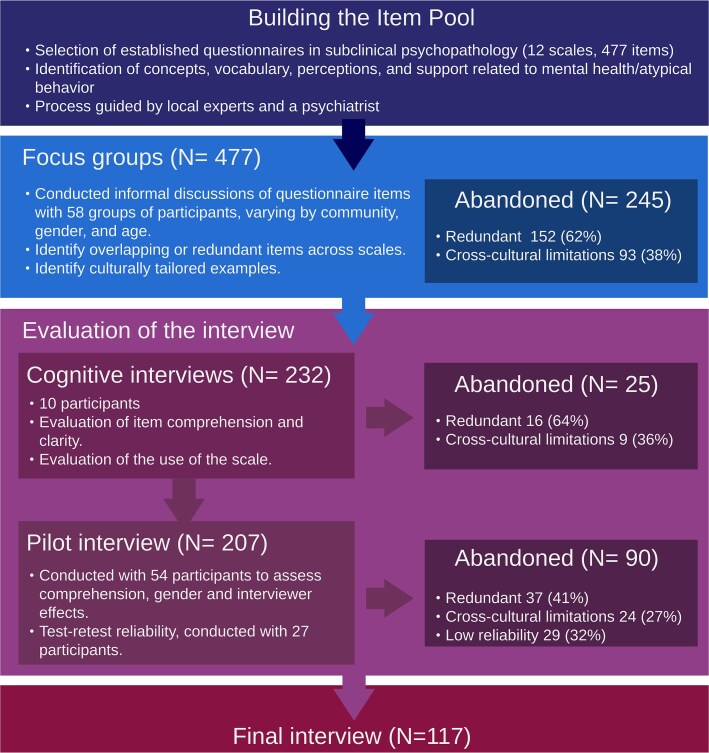
Flowchart and pipeline of the interview, numbers in parentheses (e.g. *N* = 477) refer to the number of items.

Figure 1 summarizes the pipeline, and the workflow outlined below follows the World Health Organization (WHO) recommendations and guidelines and cross-cultural literature on self-reported measures [26, 27].

### Building the item pool

#### Scales selection

We identified prompts from existing questionnaires used to study subclinical psychopathology and personality. The 12 scales we used are the Autism Spectrum Quotient [28], the Broad Autism Phenotype Questionnaire [29], and the Comprehensive Autistic Trait Inventory [30] for ASD. For schizotypal personality traits, we used the Wisconsin Schizotypy Scales [31] and the Schizotypal Personality Questionnaire [32]. For OCD, we used the Obsessive-Compulsive Inventory-Revised [33], for ADHD, the Adult ADHD Self-Report Scale (ASRS, [34]), and for anxiety, the generalized anxiety disorder (GAD-7) scale [35]. For behavioral problems like substance abuse, interpersonal conflict, and sleep problems, we used the International Mental Health Assessment [36]. We also employed the Tsimaneʼ depression scale [8]. Additionally, for general personality, we used the Big Five Inventory [37, 38] that has been previously used with the Tsimaneʼ [24] and the Big Two survey [39], argued to be more appropriate for non-Western, Educated, Industrialized, Rich, and Democratic (WEIRD) populations.

When available, we used validated Spanish translations (e.g. [40–42]) and translated other items ourselves. In total, the aggregation of all scales resulted in 477 items.

#### Discussions with local and external experts

The first goal was to better understand Tsimaneʼ concepts and the language used to describe personality traits and mental health-related topics. Our team included three Tsimaneʼ research assistants (including co-authors A.B.C. and A.V.T.), each with over 10 years of experience working in virtually all Tsimaneʼ communities, who provided crucial local knowledge, as well as a psychiatrist (C.V.D.D.), who conveyed the intended use and meaning of the scales in Global North contexts.

Initial discussions focused on extreme phenotypes associated with various psychopathologies, as well as unusual personality traits more broadly. Subsequently, we reviewed all 477 items in the original scales. Research assistants identified questions that seemed relevant to the Tsimaneʼ community but rarely encountered in practice, highlighted difficult concepts, and helped define examples and idioms of distress, metaphors, or explanations that would make questions more comprehensible. These discussions revealed some atypical behaviors and prompted conversations about people’s perceptions and local approaches to mental health and behavioral differences, including acceptance, stigma, and practical strategies for managing these traits.

### Focus groups

We organized focus groups to expand on these topics. Focus groups were conducted predominantly with same gender (We preferred same-gender groups because participants are more comfortable discussing sensitive topics in the presence of their same gender. Whenever possible, we organized groups from the same generation, as older individuals often dominate conversations, leaving less room for younger participants to speak.) participants and included young, older, and mixed-age groups. Each group consisted of two to seven individuals, allowing for in-depth discussions in a comfortable setting. In total, we interviewed >250 individuals in 58 different focus groups across three different communities, each with varying levels of market integration, subsistence lifestyle, access to schooling, and local ecological factors (e.g. presence of a river and proximity to the main town). Focus groups lasted between 1 and 1.5 hours and were initially structured around open-ended prompts (Supplementary Materials SM1). Notably, prompts about local healers and issues that Western medicine cannot solve proved very useful to elicit conversations about mental health. Direct questions about mental health often failed to elicit meaningful responses. Participants would either appear unsure of what we meant or say that those problems do not happen in their community or to them. In contrast, asking when and why people sought help from local healers led to rich discussions. Many explained that they turn to healers when doctors have not been effective, or when the nature of the problem is perceived to be beyond the reach of “Western medicine.” These conversations often included detailed descriptions of symptoms, behaviors, and community responses to individuals in distress, which helped us identify culturally meaningful expressions of what might be labeled psychiatric traits. Several items in our final instrument were grounded in these narratives and examples.

Focus group discussions were conducted by C.S., A.B.C., and A.V.T.; C.S. set the main topic of conversation and questions in Spanish, which were then immediately translated into Tsimaneʼ by A.B.C. and A.V.T. We aimed to cover the topics of the 477 items from the questionnaires in a casual conversational manner. We provided examples of the various traits using locally relevant scenarios. Particular emphasis was placed on exploring what extreme traits stood out as unusual within the community. Often, participants identified individuals fitting some description. When this occurred, we asked follow-up questions to gather more detailed information about these individuals but also encouraged participants to think of others with similar traits and to provide additional descriptions of the individual, their environment, and their personal history. This method allowed us to identify individuals who displayed particular traits while ensuring that definitions of distress or impairments were based on local perception rather than imposing external diagnostic standards.

Using insights gathered from the discussions with research assistants and focus groups, we adapted and translated specific items into the Tsimaneʼ language. Redundant items were merged to reduce the interview length (see Supplementary Materials SM10). All items were transformed into questions, given the impossibility of doing a self-assessment survey in a population with low literacy levels. Frequency terms like “often” or “sometimes” were removed from the questions due to confusion in the interpretation. Additionally, concrete examples were provided for most questions, specifically tailored to fit Tsimaneʼ day-to-day activities. For example, without any concrete examples, research assistants and participants considered questions like “I see myself as someone who is easily distracted” to be too vague. As we sampled a broad range of contexts during our focus groups, we opted for examples that resonated more broadly with all participants. For example, building houses or harvesting/maintaining fields are ubiquitous tasks all over the territory.

Items that generated confusion, systematic misunderstanding, or proved too abstract to be anchored in culturally meaningful examples were abandoned. These encompassed concepts without clear lexical equivalents (e.g. “relaxing/time off”), items reliant on numeracy or literacy skills (e.g. dates, reading a story), and those involving figurative or idiomatic language (e.g. “reading between the lines”). A complete list of abandoned items is provided in Supplementary Materials SM8–SM10. When the underlying construct was already represented by a clearer item, we retained the comprehensible alternative and dropped the problematic item.

After extensive discussion, we determined that a 5-point Likert scale (“Never”, “Almost never”, “Sometimes”, “Almost always”, and “Always”) was most appropriate. Two extra options (“Did not answer” and “Did not understand the question”) were added to the scale. The first is used when participants choose not to respond or time constraints require skipping the question, and the second is for when participants could not understand the question despite rephrasing or additional examples. Following these adjustments, the questionnaire was reduced from 477 to 232 items (see Fig. 1). Translations were reviewed through a rigorous process involving initial translation and back-translation by a research assistant not familiar with the questions. Discrepancies were resolved through discussion.

### Evaluation of the interview

We conducted two rounds of evaluation to test the content comprehension and reliability of the interview. First, we conducted cognitive interviews with 10 volunteers (60% female) to assess whether the questions effectively captured the intended information [43]. We asked each participant to explain their reasoning for answering each question. Specifically, we prompted them with: “*Can you tell us in your own words what that question was asking about and why you gave us that answer in the scale*?” This occasion was also used to train research assistants on how to deliver the prompts and handle participants’ questions and concerns. It also served as a troubleshooting session for an electronic version of the survey. This led to the exclusion of 25 additional questions, leaving the interview to be 207 items long.

Second, we evaluated reliability. To address participant fatigue and reduce costs, we divided the 207-item interview into three subsets of 69 questions each (the full interview lasted 2.5–3.5 hours, while each subset lasted ~45 minutes–1 hour). A convenience sample of 54 Tsimaneʼ adults completed one subset of 69 questions to assess the Likert scale used, overall comprehension, and potential gender and interviewer effects. Additionally, 27 participants completed the same subset twice (8 for version 1, 10 for version 2, and 9 for version 3), with an average interval of two weeks between sessions, to evaluate test–retest reliability. We also created two semi-randomized versions of each subset to evaluate potential order effects.

### Ethics

All research protocols were approved by the ethics committee of the University of Zurich (#22.9.11) and by the Tsimaneʼ Government (Gran Consejo Tsimaneʼ). Before commencing research in a community, we held a meeting to describe the objectives of the study and answer any questions of prospective participants as well as to get the community’s approval. Verbal consent was obtained during the initial recruitment of study participants and immediately before participation. All participants of the focus groups and interviews received compensation proportional to their time.

## RESULTS

### Evaluation of the interview

#### Cognitive interviews

The 10 cognitive interviews, covering 232 items, lasted between 3 and 5 hours, with several breaks and refreshments provided to prevent fatigue. At this stage, 25 questions were excluded. Some were redundant with items already included, while others were removed due to persistent comprehension problems despite adjustments in wording and the addition of examples (i.e. cross-cultural limitations). For instance, the item *“I have worried that people on other planets may be influencing what happens on Earth”* was adapted to refer instead to “outside forces,” evoking folk spirituality about the specific type of spirits. However, even with this adaptation, the item continued to generate confusion and was often conflated with religious beliefs (e.g. references to Jesus being in all of us and everywhere). Similarly, the item *“I engage in certain repetitive actions when I feel stressed”* proved problematic, as the concept of “feeling stressed” lacked a direct equivalent. While participants recognized behaviors such as nail-biting or pulling grass, they did not associate these actions with distress. Consequently, the requirement to explicitly link repetitive behaviors with worry or stress was dropped. In contrast, items that showed only partial misunderstanding were revised through changes in wording and the inclusion of clearer, culturally appropriate examples.

#### Pilot interview

##### Scale use

We evaluated the distribution of responses across the Likert scale. This is a necessary step to evaluate the use of the scale and check the number of non-responses (lack of understanding or refusal to answer). We computed the range, variance, standard deviation, and coefficient of variation by question. For a 5-point Likert scale, the range can be 0 (no variability, everyone gave the same response) to 4 (maximum variability). An intermediate value (1, 2, or 3) indicates that some levels were skipped. Almost all questions had a range above or equal to 2, and a majority had a range equal to 4 (Supplementary Material SM2), indicating that participants used all levels of the scale for the majority of questions.

**Figure 2:**
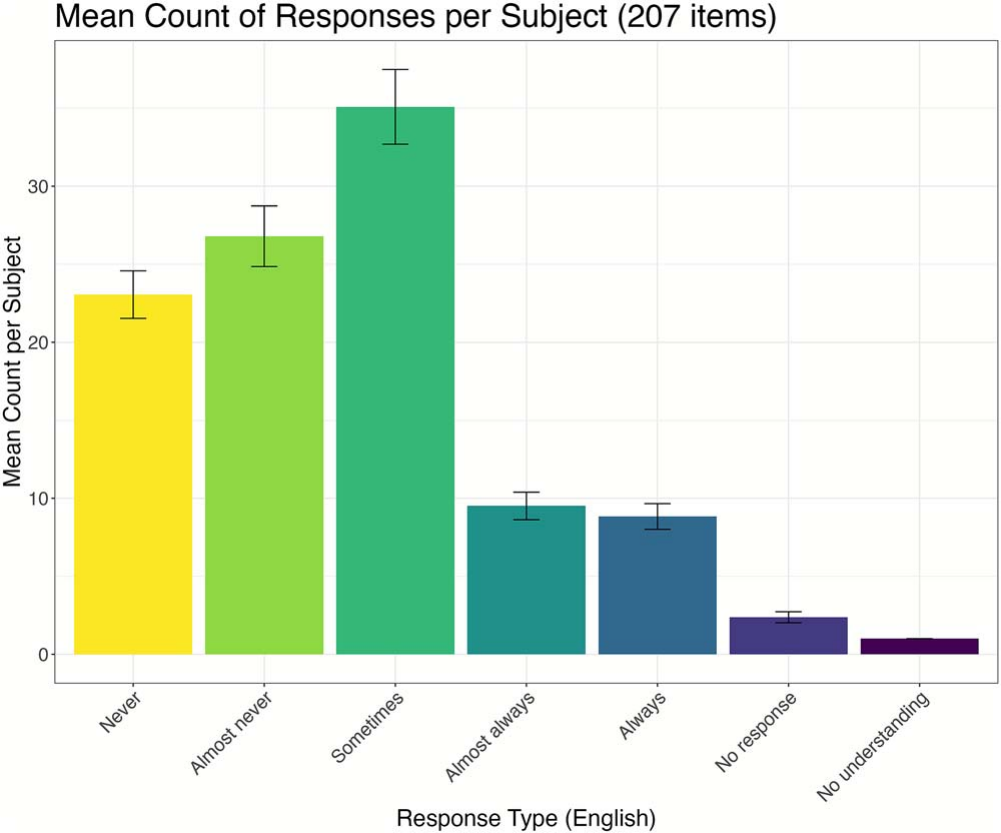
Distribution of answers to the 207-item long interview.

As shown in Fig. 2, all categories were used with very low levels of non-responses. There was a slight bias to the middle level (“sometimes”), without exceeding 50% of total responses. In addition, negative answers (“almost never” and “never”) were used more than positive ones (“almost always” and “always”). The kurtosis = 2.5 suggests a flatter-than-normal distribution; responses are distributed across multiple levels rather than clustering at the extremes or middle. The Kurtosis for the final 117-item interview was 2.6. This indicates that participants are not overly clustering in the middle, a potential problem of using Likert scales that can reflect a lack of understanding [44].

Finally, the two semi-randomized versions allowed us to evaluate potential order effects. The response distributions were very similar, suggesting no major order effects (see Supplementary Material SM3). In addition, we observed similar distributions of answers between men and women and among the three interviewers, suggesting consistency in how the instrument was understood and administered (Supplementary Material SM4). Some questions showed some differences. These items were closely examined to determine whether observed differences reflected true trait variation or variation in the question framing by gender or interviewer (examples provided in Supplementary Material SM5).

##### Test retest

**Figure 3:**
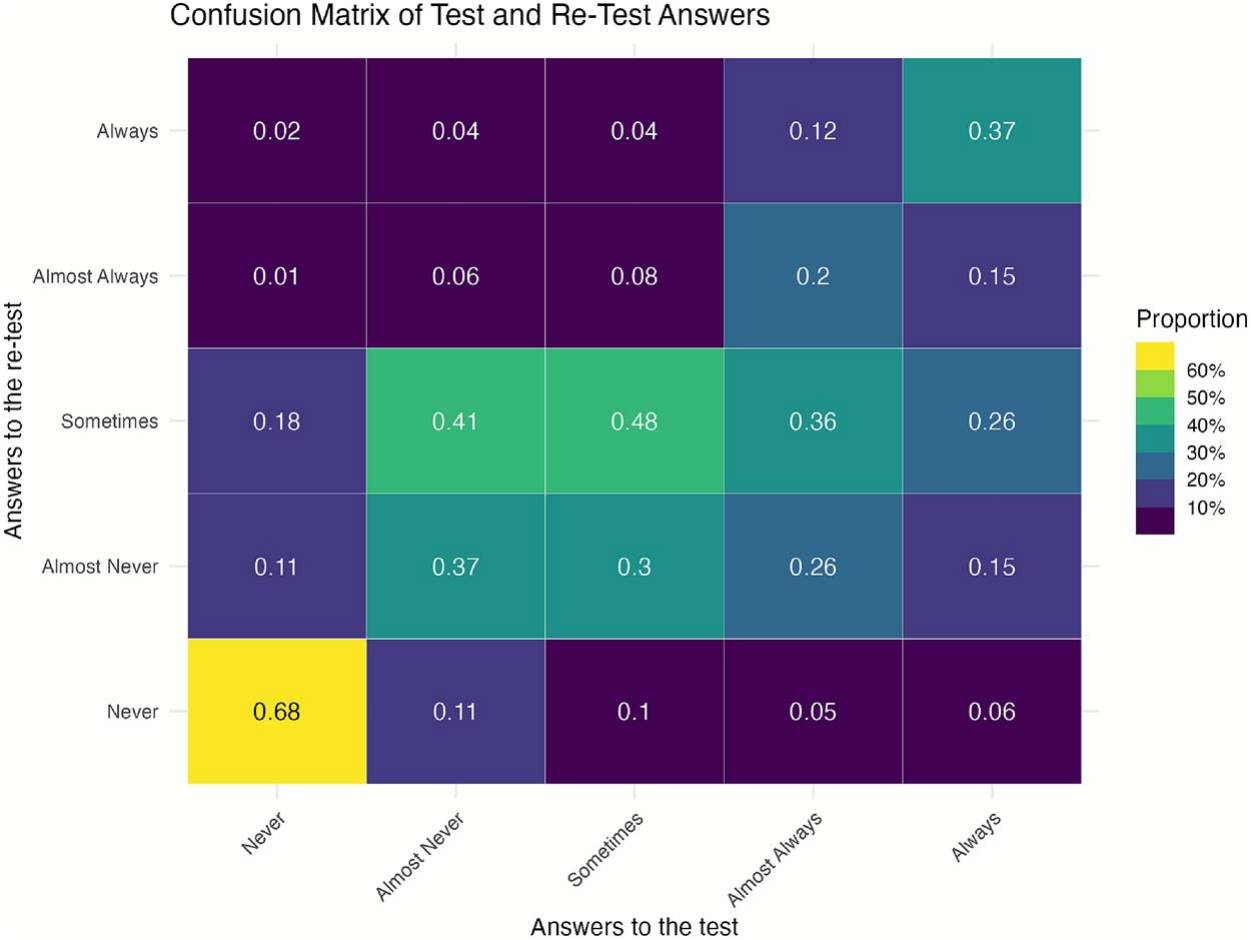
Confusion matrix for test/Re-test questionnaire representing how responses from an initial test align with responses from a retest.

We use test–retest to evaluate the consistency and comprehension of the instrument over time, with the underlying assumption that the questions and traits evaluated should remain stable, particularly over a short interval (in this case, 2 weeks). In the confusion matrix shown in Fig. 3, perfect agreement (proportion of responses that remained the same between test and retest) can be observed in the diagonal, from 0.68 to 0.37. For example, the response “*never*” showed the strongest consistency (0.68). Off-diagonal values indicate changes in responses between test and retest. The highest tendency to shift was from “*almost never” to “sometimes”* (0.41), suggesting fluidity between these adjacent responses. Shifts at the extremes were low, e.g. 0.11 from *“never”* to *“almost never”* and *“almost never”* to *“never”; 0.15/0.12* from “*always”* to *“almost always”* and *“almost always”* to *“always*.” This pattern suggested that participants understood the questions consistently, with most variation occurring between adjacent categories rather than at the extremes. To address this, dedicated sessions of training were devoted to explaining the use of the scale between adjacent answers (particularly between “*almost never” and “sometimes”).*

We used weighted Cohen’s Kappa to identify which items were more reliable. Weighted Cohen’s Kappa is more appropriate for Likert scales, accounting for the ordinal nature of the responses, where smaller shifts (e.g. “2 ↔ 3”) are less penalized than extreme shifts (e.g. “1 ↔ 5”). Forty-six percent of answers had a *perfect agreement* (same answers on the scale), and 80% had a *perfect or very high agreement*, same answer or within one adjacent response on the scale. The range for weighted kappa (0.43–0.52) supports a moderate level of agreement.

We identified the questions with the lowest kappa (below or equal to zero), indicating poor agreement (*N* = 71). Some of the questions revealed issues with reverse-coding; for example, participants responded “*never*” to one item and “*always*” to the second time. Reverse coding issues primarily arose for prompts where the wording, particularly the use of negatives, caused confusion. For instance, the prompt “Reading non-verbal cues (e.g. facial expressions, body language) is difficult for me” led to varying interpretations, as some participants interpreted it as something they are good at (interpreting non-verbal cues), rather than focusing on the “difficult for me” part. All poor agreement questions were further investigated and discussed, and were considered as candidates for removal from the final instrument (see Supplementary Material SM9).

#### Cognitive interview with “non-adapted” items

To illustrate the need to adapt items and to train interviewers, we asked a seasoned research assistant, unfamiliar with this specific interview but with more than a decade of experience conducting anthropological surveys and data collection, to conduct cognitive interviews on a subset of the original Spanish items. For this, the interviewer translated the Spanish items into Tsimaneʼ during the interview without any prior training. We ensured that he understood the intended meaning of each question and was allowed to recast or add examples, mimicking a normal interview process.

A total of 22 participants (68% female) responded to a subset of 20 questions, consisting of two items from each subscale of the AQ and Big Five Inventory (see Supplementary Material SM6). We evaluated each response in two areas: first, whether the participants understood the overall meaning of the question, and second, whether the meaning of their answer was accurately reflected in their selected answer on the scale. So, each question was categorized as “understood and answer matches scale”, “understood and answer does not match scale,” or “not understood.”

On average, 37% of responses were categorized as “understood and answer does not match scale”, followed by 25% categorized as “not understood.” Only 36% of questions were in the “understood and answer matches scale” category. Many of the “understood and answer does not match scale” responses stemmed from issues related to reverse coding. There was substantial variation depending on the specific question (see Supplementary Material SM6 for more details).

For some items, such as *“I tend to notice details that others do not”* and “*I often notice small sounds when others do not”,* none of the participants correctly understood the intended meaning, despite the interviewer confirming that he understood the correct meaning of the questions. All of the participants interpreted these two items as associated with one-time supernatural experiences (e.g. seeing a ghost).

### Overview of the final survey

**Figure 4:**
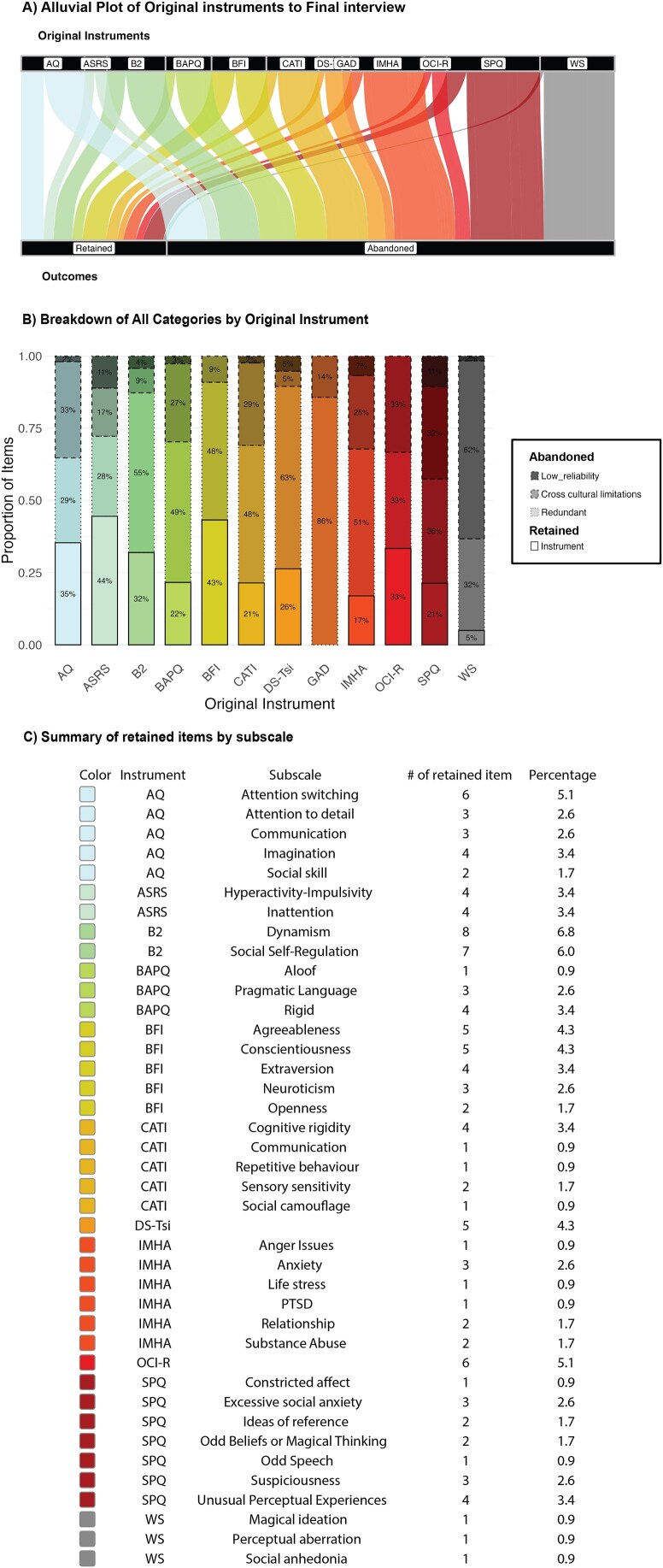
Summary of the final instrument. (A) Alluvial plot showing the proportion of items retained (instrument) or abandoned (cross-cultural limitations, redundant, low reliability). (B) Stacked bar plot; (C) table summarizing the composition of the final 117-item interview with percentages by subscales of the original instruments. Abbreviations: ASRS = adult ADHD self-report; AQ = autism quotient; B2 = cross-cultural big two; BAPQ = broad autism phenotype questionnaire; BFI = big five inventory; CATI = comprehensive autistic trait inventory; DS-Tsi = depressive symptoms (Tsimaneʼ); GAD = generalized anxiety disorder; IMHA = international mental health assessment; OCI-R = obsessive-compulsive inventory–revised; SPQ = schizotypal personality questionnaire; WS = Wisconsin scales of vulnerability to psychosis–brief.

For the final survey, we prioritized retaining questions showing very good comprehension and agreement, and showing variation in responses. The evaluation process presented above resulted in the exclusion of 90 additional questions (see Fig. 1). The final instrument consists of 117 questions (see Figure 4A for summary by scale and Figure 4B and 4C for summaries by subscales, see Supplementary Material SM7 for complete list of questions), taking roughly 1 hour to complete.

## DISCUSSION

Our study provides a step-by-step methodology to assess subclinical psychopathological and personality traits in small-scale societies. We developed a culturally adapted instrument for the Tsimaneʼ population by adapting existing questions from diverse sources through extensive focus groups, cognitive interviews, and input from local research assistants. The final survey of 117 items shows comprehension and variation of the different questions among adult participants. The present design provides the necessary groundwork for future studies examining construct validity, internal consistency, and external validity more formally, and to extend this approach to other small-scale or underrepresented populations.

Traditional psychiatric tools are designed and tested to be reliable and generalizable across individuals and populations. However, they are often designed for the Global North and can fail to capture the nuanced ways in which traits manifest in other cultural settings. Here, we tried to overcome these limitations by incorporating culturally relevant and concrete examples and idioms of distress and converting self-report questions into an interview. Our survey also includes qualitative information to explore how traits are experienced and perceived locally.

This study highlights the critical role of collaboration with local experts (such as experienced research assistants) and communities. Their input was essential for ensuring cultural relevance, participant trust, and accurate interpretation of results. Our research also acknowledges the important role of local healers and traditional practices in shaping how mental health is understood and expressed within the community. Many Tsimaneʼ repeatedly emphasized that they turn to traditional healers and remedies for mental health concerns, which they often perceive as inadequately addressed by Western medicine. Discussions about when and why individuals sought help from healers produced detailed, culturally meaningful accounts of symptoms, behaviors, perceived causes, and community responses. These narratives provided insight into how “extremes” of experience are locally recognized and when they become a source of concern. Future efforts could benefit from integrating traditional healing practices into broader interventions and therapies [45], as in our study, they proved deeply informative for identifying distinct mental health phenotypes.

Given the logistical challenges of fieldwork, the present study was deliberately designed to maximize coverage of diverse traits across constructs and to ensure that items were culturally and linguistically comprehensible. Because the questionnaire was deliberately fragmented into different subsets to reduce interview burden during the evaluation stage, participants did not complete full scales, which prevented the use of conventional reliability metrics such as Cronbach’s α. Instead, we employed item-level correlation approaches as a first step toward examining reliability and discriminant validity. While these analyses did not reveal strong clustering of items by meta dimension (e.g. ASD, Personality…), this outcome reflects the design’s limitations given its primary intention rather than a lack of construct validity (see Supplementary Material SM11). An important open question, which our current design and sample sizes could not address, is whether the dimensional structure of these traits aligns with descriptions in the literature and whether it is consistent across different subgroups of the population (e.g. variation by market-integration). One of our ultimate goals is to quantify subclinical psychopathological traits in order to test evolutionary hypotheses about the origins and maintenance of psychopathology [3–5, 12, 46].To move toward this goal, a key next step will be to formally evaluate the validity of the instrument, which will require larger samples and complete within-person responses across all items. Studies in other small-scale societies should replicate this methodology to explore the universality and variability of these traits. Long-term research could investigate how traits change over time and reflect upon the items that got lost in translation and why.

We acknowledge that certain rare traits might be underrepresented due to sample size or self-participant selection. Some individuals with severe atypical behaviors (e.g. extreme social withdrawal, which was described in our focus groups) may not have participated in our interviews, potentially skewing the results.

## CONCLUSIONS AND IMPLICATIONS

Finally, one of our core goals with this paper is to support and encourage others conducting culturally-grounded instrument adaptation, particularly in small-scale or underrepresented societies. This work is often slow and painstaking, but we hope our experience can help guide future efforts. Based on what we learned, we recommend grounding the adaptation process in ethnography through participant observation, focus groups, and sustained collaboration with local experts before formalizing any instrument. Equally important is to “meet halfway” by engaging in detailed discussions with community members to ensure that items reflect locally meaningful behaviors, symptoms, and idioms of distress relevant to the research focus. Providing concrete, culturally relevant examples tailored to diverse contexts (e.g. gender, age, market integration) can further support comprehension. Iterative piloting through cognitive interviews is critical, allowing researchers to refine wording and framing before formal testing. Particular caution is warranted with reverse-coded items, which were especially prone to misinterpretation in our setting: these should be avoided when possible, or carefully flagged, rephrased, and checked through interviewer follow-up (e.g. confirming that a response of “never” to “Is it hard to understand jokes?” truly meant that understanding jokes was never difficult). Investing in thorough interviewer training, including rephrasing protocols and curated examples, but also, if possible, including them since the conception of the project, can help ensure consistent administration across challenging items. Finally, we encourage transparent documentation of all adaptations, exclusions, and decision points so that others can replicate, evaluate, and further adapt the process.

## Supplementary Material

supmatall_eoaf033

## Data Availability

Due to ethical considerations the data are not publicly available. Access may be granted upon reasonable request by contacting the first and last author.
