# Supplementary material for: Assessing subclinical psychopathological and personality traits in a small-scale subsistence society
Source: Evol Med Public Health. 2025 Nov 20;13(1):413–23. doi: 10.1093/emph/eoaf033 (PMC12758380; doi:10.1093/emph/eoaf033)
Supplement: supmatall_eoaf033 [file supmatall_eoaf033.pdf]

Supplementary Materials for Assessing Subclinical Psychopathological and Personality Traits in a Small-Scale Subsistence Society

Table SM1. List of the most useful topics to discuss psychopathological traits for focus groups

What types of diseases can Western medicine not cure or is ineffective for
Reasons to seek the local healer
Use of plants (known to have healing properties)
Methods of maintaining land and harvesting crops (or other subsistence-related activities)
Nocturnal activity (problems sleeping, doing activities alone at night)
Withdrawal from community social events or rituals
Markers of atypical development in children (delayed (or no) onset of speech and/or walking)

Table SM2. Number and percentages of questions by range for the two evaluation stages of the interview.

range	0	1	2	3	4
Questions 207 (count)	1(>1%)	2 (1%)	36 (17%)	77 (37%)	91 (44%)
Questions 117 (count)	1 (>1%)	1 (>1%)	15 (13%)	43(37%)	57 (49%)

Figure SM3. Distribution of answers as a function of the semi-randomized version of each subset

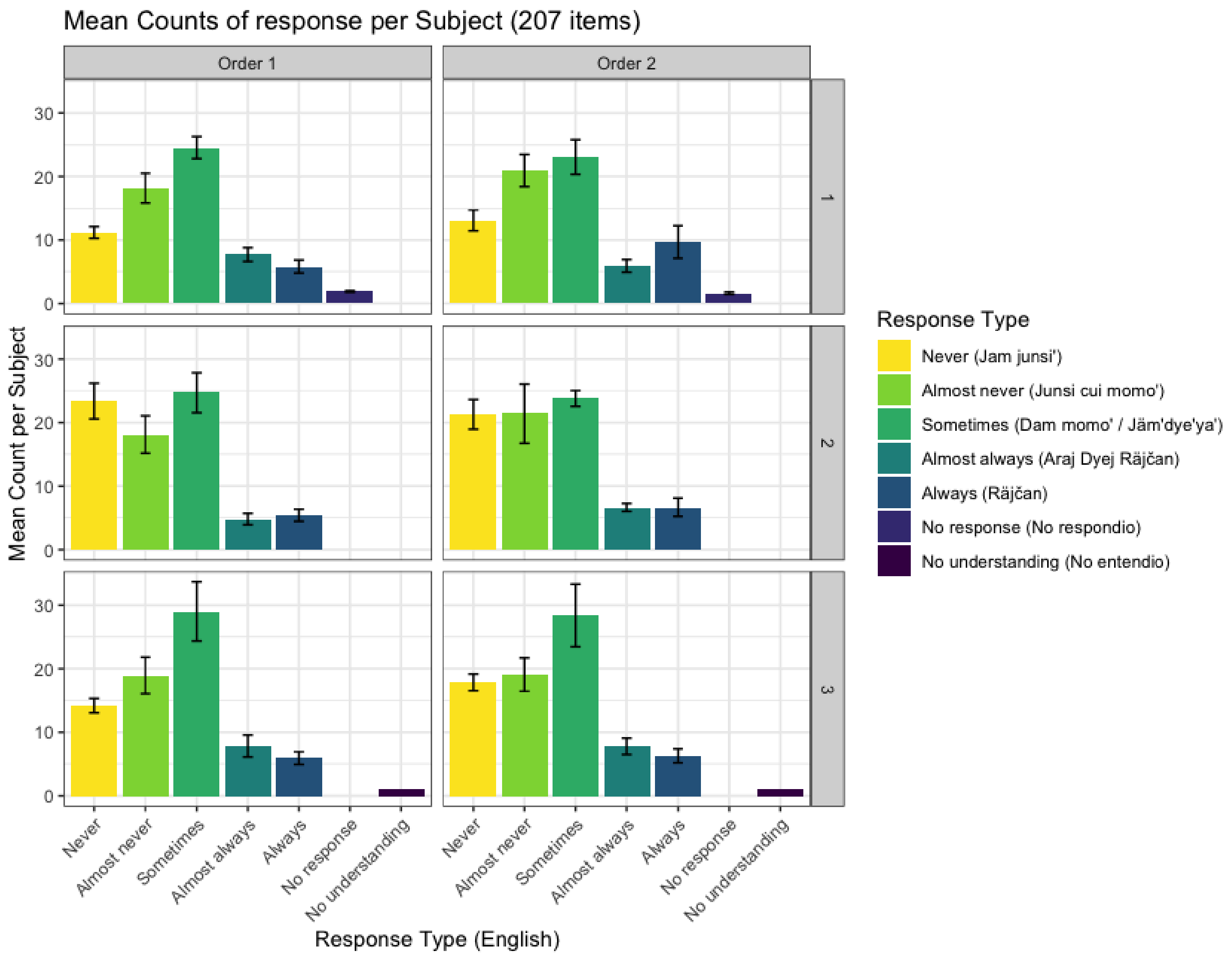

Figure SM4. Distribution of responses by gender and interviewer

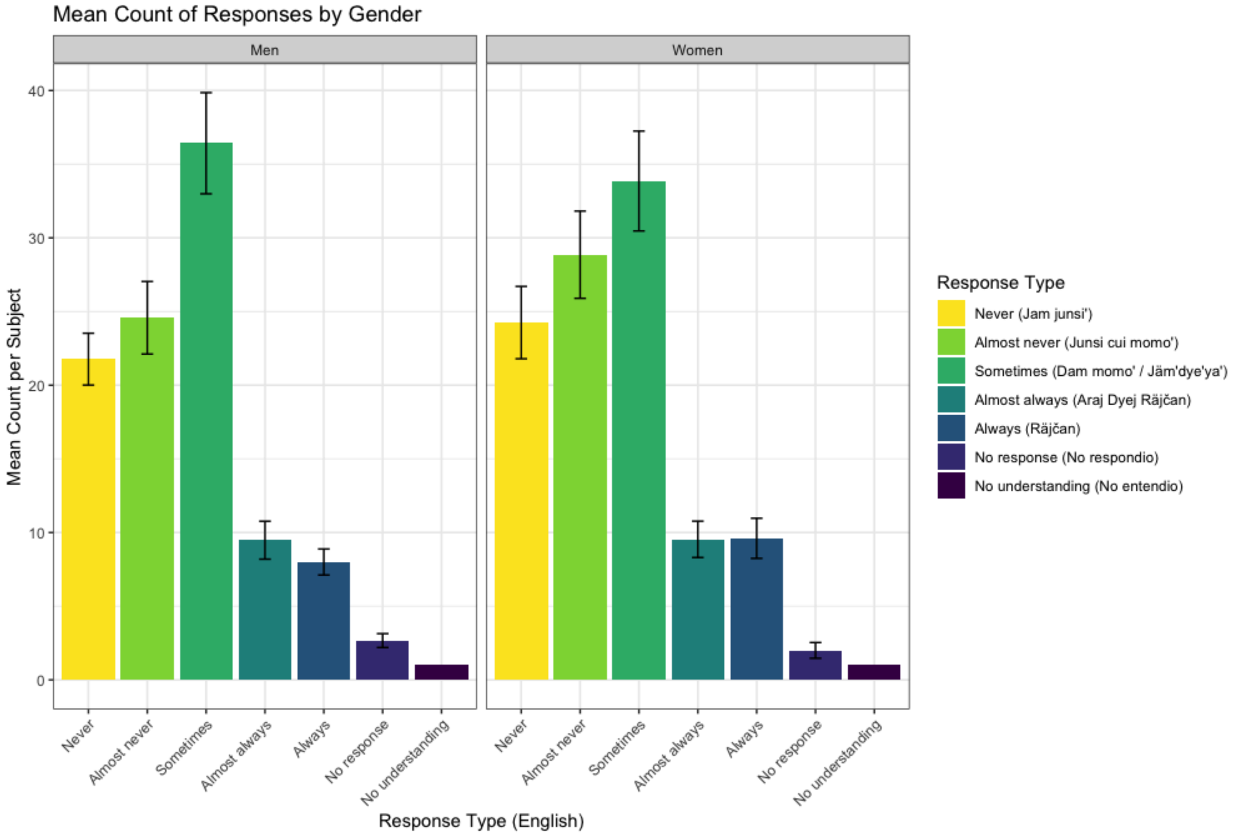

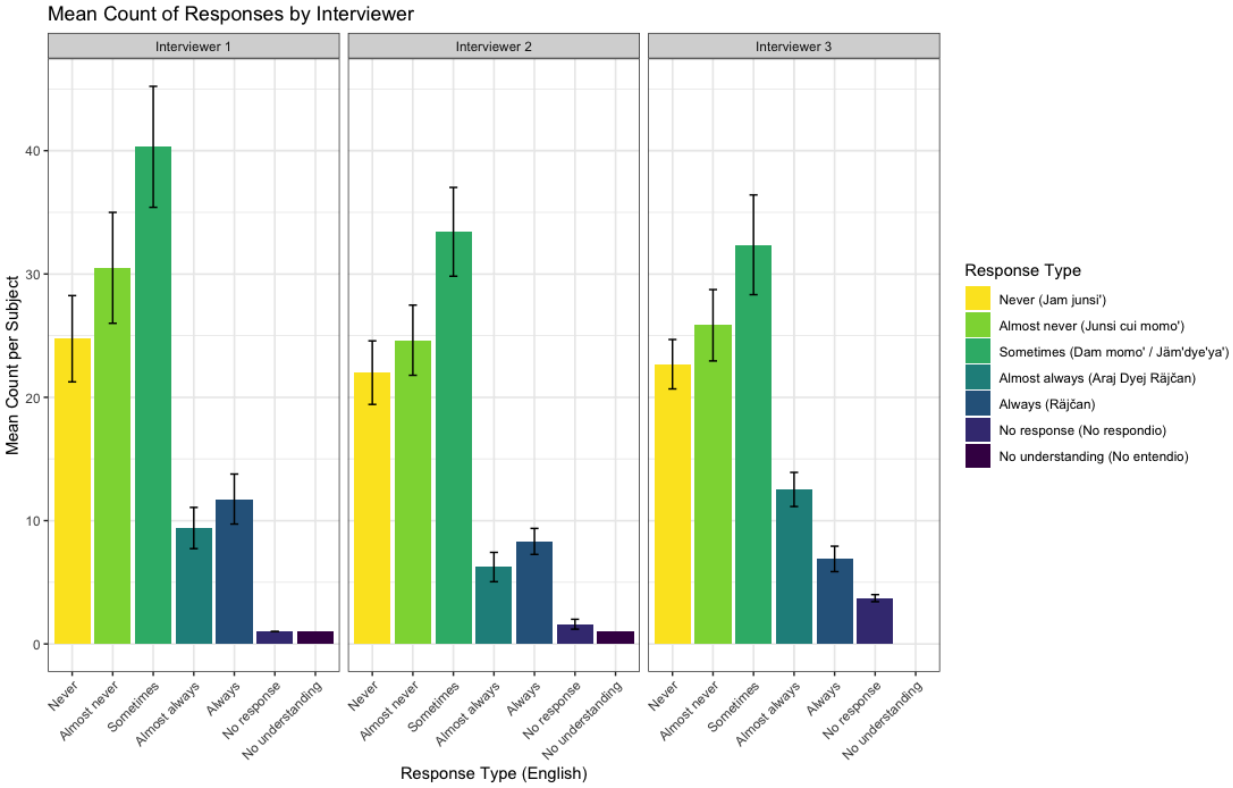

Figure SM5. Questions showing gender or interviewer variation.

A. Distribution of the question: “In the last month, I had difficulty breathing (not due to a medical condition)”.

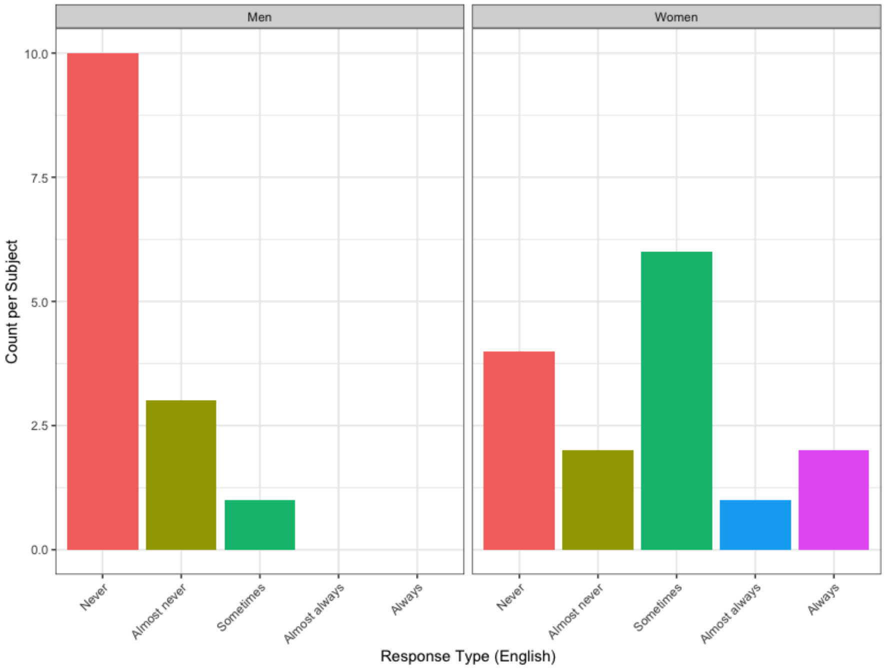

B. Distribution of the question: “I have certain habits that I find difficult to stop (e.g. biting/tearing nails, pulling strands of hair)”.

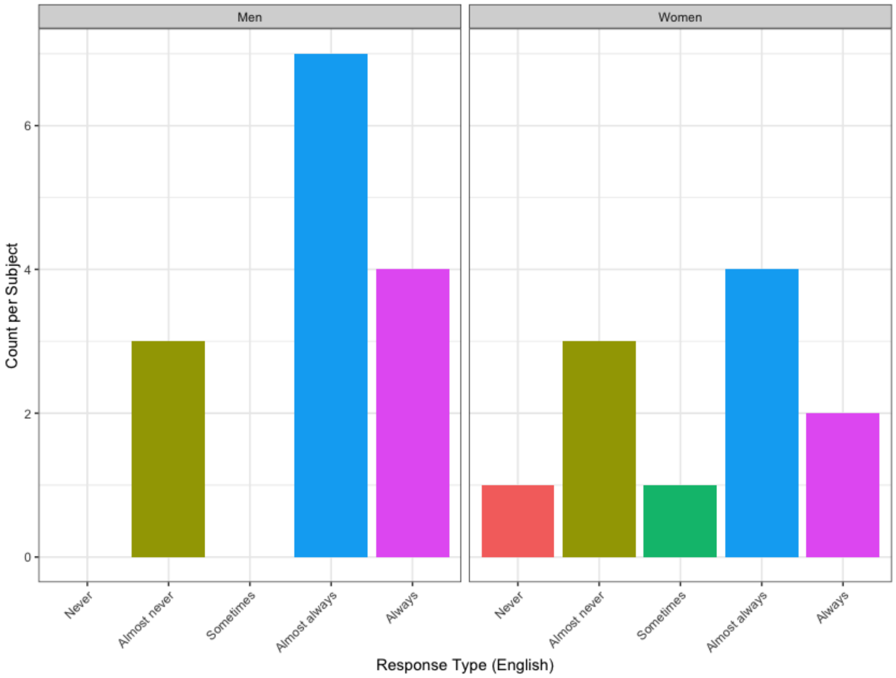

C. Distribution for the question: " I tend to avoid eye contact when conversing with others".

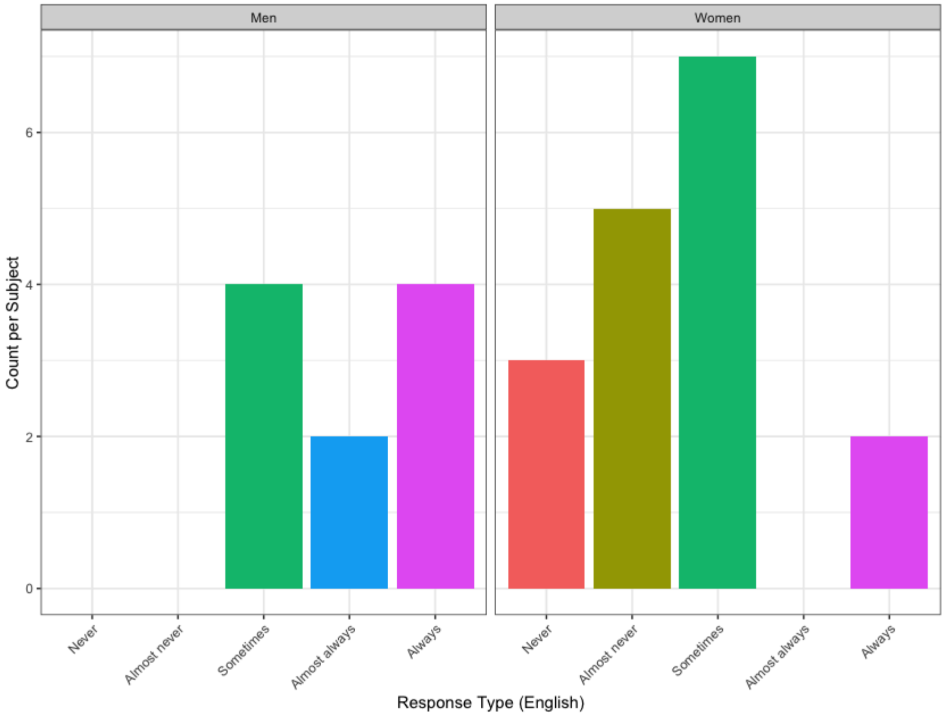

D. Distribution for the question: "I prefer to be alone rather than with others".

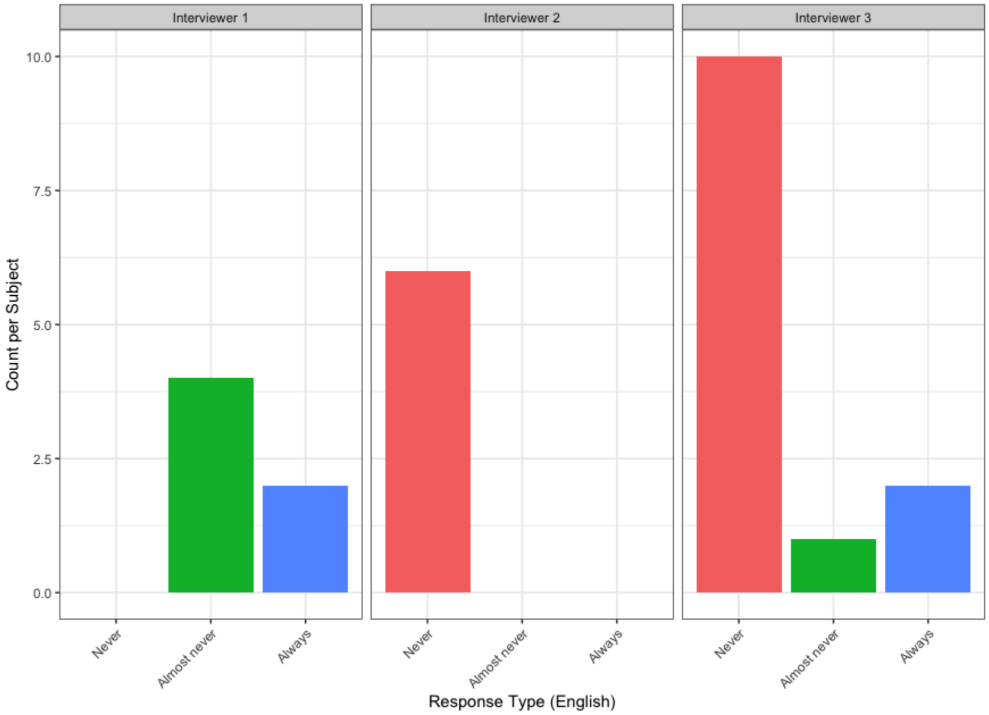

Table SM6. List of items used for the cognitive interviews of non-adapted items and their associated answers.

Abbreviations: ASRS = Adult ADHD Self-Report; AQ = Autism Quotient; B2 = Cross-Cultural Big Two; BAPQ = Broad Autism Phenotype Questionnaire; BFI = Big Five Inventory; CATI = Comprehensive Autistic Trait Inventory; DS-Tsi = Depressive Symptoms (Tsimane'); GAD = Generalized Anxiety Disorder; IMHA = International Mental Health Assessment; OCI-R = Obsessive-Compulsive Inventory–Revised; SPQ = Schizotypal Personality Questionnaire; WS = Wisconsin Scales of Vulnerability to Psychosis–Brief.

Scale	Subscale	Question English	Question Spanish	% understood and answer matches scale	% understood and answer don't match scale	% not understood
AQ	Attention switching	I tend to have very strong interests which I get upset about if I can't pursue.	Tiendo a tener intereses muy fuertes, y me molesta si no puedo dedicarles tiempo.	0%	0%	100%
AQ	Attention switching	I find it easy to do more than one thing at once	Me es fácil hacer más de una cosa a la vez.	59%	36%	5%
AQ	Attention to detail	I tend to notice details that others do not	Tiendo a notar detalles que otras personas no.	0%	0%	100%
AQ	Attention to detail	I often notice small sounds when others do not	Frecuentemente puedo oír débiles sonidos cuando otros no pueden.	0%	9%	91%
AQ	Communication	I know how to tell if someone listening to me is getting bored	Puedo reconocer si alguien que me escucha se está aburriendo.	18%	27%	55%
AQ	Communication	I am often the last to understand the point of a joke	En general, soy el último en entender un chiste.	50%	41%	9%
AQ	Imagination	I don't particularly enjoy reading fiction	No me agrada particularmente la lectura de ficción.	54%	32%	14%
AQ	Imagination	When younger, I enjoyed playing games involving pretending with other children	Cuando era joven, disfrutaba jugar juegos de roles con otros niños (por ejemplo: mamá y papá, vaqueros, etc.).	68%	32%	0%
AQ	Social skill	I find social situations easy	Las situaciones sociales me son cómodas.	27%	59%	14%
AQ	Social skill	I am a good diplomat	Soy un buen mediador.	27%	64%	9%
BFI	Agreeableness	I see myself as someone who starts disputes with others	Me veo a mi mismo como alguien que inicia disputas con los demás	32%	64%	4%
BFI	Agreeableness	I see myself as someone who is generally trusting	Me veo a mi mismo como alguien que es generalmente confiado	41%	54%	5%
BFI	Conscientiousness	I see myself as someone who is meticulous at work	Me veo a mi mismo como alguien que es minucioso en el trabajo	36.50%	36.50%	27%
BFI	Conscientiousness	I see myself as someone who is easily distracted	Me veo a mi mismo como alguien que se distrae con facilidad	64%	22%	14%
BFI	Extraversion	I see myself as someone who tends to be quiet	Me veo a mi mismo como alguien que tiende a ser callado	55%	36%	9%
BFI	Extraversion	I see myself as someone who is outgoing	Me veo a mi mismo como alguien que es extrovertido, hablador	32%	45%	23%
BFI	Neuroticism	I see myself as someone who cares a lot about things	Me veo a mi mismo como alguien que se preocupa mucho por las cosas	41%	41%	18%
BFI	Neuroticism	I see myself as someone who is temperamental, moody	Me veo a mi mismo como alguien que es temperamental, de humor cambiante	59%	32%	9%
BFI	Openness	I see myself as someone who values the artistic, the aesthetic	Me veo a mi mismo como alguien que valora lo artístico, lo estético	45%	50%	5%
BFI	Openness	I see myself as someone who prefers jobs that are routine	Me veo a mi mismo como alguien que prefiere trabajos que son rutinarios	22%	73%	5%

Table SM7. Complete list of “original” items retained in the final questionnaire

All items were rephrased as questions rather than self-assessment statements. Frequency terms such as *“often”* or *“sometimes”* were removed to improve clarity in the Tsimane’ version. Items that were too similar, either before or after translation/adaptation, were merged. Thus, the “original items” listed below may correspond to multiple items from different scales. Whenever possible, items were framed using concrete behaviors rather than vague prompts using examples based on focus groups and interviews. “Translation in English” is provided for illustration; it may not reflect the literal wording, but rather approximate the intended meaning of the items.

Notes:

Items 20 and 21 (AQ41 - “I like to collect information about categories of things") were split into two distinct questions during adaptation. Discussions with local research assistants and focus groups revealed that respondents interpreted this prompt in two very different ways:

- As “abstract information gathering” (e.g., someone who liked memorizing all players and teams of a football league).
- As “physical object collecting”, often involving unusual or rare items (e.g., feathers, bones, artifacts, or stone tools, or weird-shaped stones, and often these items seem to be displayed at home.

Abbreviations: ASRS = Adult ADHD Self-Report; AQ = Autism Quotient; B2 = Cross-Cultural Big Two; BAPQ = Broad Autism Phenotype Questionnaire; BFI = Big Five Inventory; CATI = Comprehensive Autistic Trait Inventory; DS-Tsi = Depressive Symptoms (Tsimane’); GAD = Generalized Anxiety Disorder; IMHA = International Mental Health Assessment; OCI-R = Obsessive-Compulsive Inventory–Revised; SPQ = Schizotypal Personality Questionnaire; WS = Wisconsin Scales of Vulnerability to Psychosis–Brief.

#	Instrument	Subscale	# in original scale	Question	Translation equivalent in English	Useful examples	Comments
1	ASRS	Inattention	1	How often do you have trouble wrapping up the fine details of a project, once the challenging parts have been done?	Is it hard for you to wrap up work that you've already started?	For men: when building a house, building a canoe, harvesting your field, removing herbs from your field For women: when weaving a traditional purse, when weaving a floor mat/basket, harvesting your field, removing herbs from your field	The item should focus on "projects" that extend over several days. Ethnographic accounts show wide variation in completion time. For instance, the fastest canoe construction takes about two weeks with non-stop continuous work, while others may abandon the process for months, resulting in rotten wood and restarting.
2	ASRS	Inattention	10	How often do you misplace or have difficulty finding things at home or at work?	Is it hard for you to find objects?	Machete; cash; identification documents; batteries; torch/flashlight.	--
3	ASRS	Hyperactivity-Impulsivity	12	How often do you leave your seat in meetings or other situations in which you are expected to remain seated?	Do you get up from your seat in situations where you are supposed to stay seated?	Community meeting; school meeting.	It is important to solicit specific concrete examples of these situations where people show individual variation. Some people say they always leave their seats, and some say they don't.
4	ASRS	Hyperactivity-Impulsivity	16	When you're in a conversation, how often do you find yourself finishing the sentences of the people you are talking to, before they can finish them themselves?	When having a conversation, do you notice that you cut (~ interrupt) someone speaking before they are done speaking?	--	--

#	Instrument	Subscale	# in original scale	Question	Translation equivalent in English	Useful examples	Comments
5	ASRS	Hyperactivity-Impulsivity	17	How often do you have difficulty waiting your turn in situations when turn taking is required?	Is it hard for you to wait for your turn when you are waiting in line?	Waiting to receive a vaccine; waiting to obtain an ID card; waiting to collect a government subsidy.	The interviewer should first identify specific situations in which the respondent waits in line and then ask whether it is difficult for them to do so.
6	ASRS	Inattention	3	How often do you have problems remembering appointments or obligations?	Do you forget appointments or important things you have to attend?	Community work; community meeting; family visit; soccer match.	The interviewer should first identify situations where the respondent is expected to be at a specific time and place, and then ask whether they tend to forget these commitments.
7	ASRS	Hyperactivity-Impulsivity	5	How often do you fidget or squirm with your hands or feet when you have to sit down for a long time?	When you have been seated for a while, do you move your hands or feet?	Community meeting; school meeting.	The interviewer should identify situations in which the respondent has to sit for a considerable time
8	ASRS	Hyperactivity-Impulsivity	7	How often do you make careless mistakes when you have to work on a boring or difficult project?	Do you make mistakes when you have to do something boring or difficult?	For men: making arrows; carving the inside of a canoe; building a house. For women: weaving a purse; weaving a floor mat or basket; making a roof	It is useful to identify tasks perceived as boring or repetitive, where inattention may lead to injury (e.g., cutting while carving) or where errors have significant consequences for completion (e.g., mismeasuring wood for house construction, making weaving errors that require undoing work).
9	AQ	Social skill	11	I find social situations easy	Do you feel uncomfortable when you're with several people, even if you know them well?	Family visits; chicha parties; birthday parties, community meetings.	Situations should reflect common local gatherings, both family- and community-based. These examples help ground the item in everyday social life.

#	Instrument	Subscale	# in original scale	Question	Translation equivalent in English	Useful examples	Comments
10	AQ	Attention to detail	12	I tend to notice details that others do not	Do you notice details or changes in things that other people don't notice?	Specific weaving patterns in a purse; the quality of an arrow; how well a field is cleaned of roots; how well plants are maintained.	Examples should highlight subtle differences that are meaningful in the local context (e.g., craftsmanship or field maintenance) but may go unnoticed by others. The different things that people attend should be assessed during focus groups.
11	AQ	Attention switching	16	I tend to have very strong interests which I get upset about if I can’t pursue.	Do you feel a very strong interest in certain things and get upset if you can't spend time on them?	Wanting to play soccer.	Examples should reflect locally relevant activities that people enjoy and may want to do somewhat frequently and where being prevented from participation causes frustration or distress.
12	AQ	Communication	17	I enjoy social chit-chat	Do you like talking with other people?	--	--
13	AQ	Imagination	21	I don’t particularly enjoy reading fiction	Do you like listening to stories?	Old stories; legends (different from gossip).	The distinction between stories/legends and gossip should be made clear during the interview to ensure respondents understand the intended meaning.
14	AQ	Communication	31	I know how to tell if someone listening to me is getting bored	Do you notice when someone is getting bored while listening to you?	People yawning; people not paying attention.	Observable cues that indicate boredom or loss of interest come from focus groups
15	AQ	Attention switching	32	I find it easy to do more than one thing at once	Can you easily do more than one thing at the same time?	Working on different small tasks alone (e.g., tending to a fire and repairing something).	Examples should emphasize doing different solo activities, rather than in group contexts.

#	Instrument	Subscale	# in original scale	Question	Translation equivalent in English	Useful examples	Comments
16	AQ	Attention switching	34	I enjoy doing things spontaneously	Do you like doing something without having to think before doing it? You just like doing it?	Play soccer, go visit someone	Examples should be drawn from everyday activities that people can engage in with or without prior planning.
17	AQ	Communication	35	I am often the last to understand the point of a joke	Is it hard to understand jokes?	--	--
18	AQ	Attention switching	37	If there is an interruption, I can switch back very quickly	If you get interrupted, can you easily go back to what you were doing?	--	--
19	AQ	Imagination	40	When younger, I enjoyed playing games involving pretending with other children	When you were a child, did you like to play to pretend to be other people?	Pretending to be an animal; pretending to be a parent; pretending to be a character from a story.	Examples are very necessary to ensure comprehension and to capture the intended meaning of pretend play.
20	AQ	Imagination	41	I like to collect information about categories of things	Do you like collecting information just for the sake of knowing?	Names of (a lot) of bird species; soccer teams,	Examples should highlight information that is not directly useful but is remembered out of personal interest.
21	AQ	Imagination	41	I like to collect information about categories of things	Do you like collecting objects?	Animal skulls; types of feathers.	Examples should emphasize locally relevant objects that people may gather and keep for personal interest rather than for immediate practical use.
22	AQ	Attention switching	43	I like to plan any activities I participate in carefully	Do you like to think in detail about the activities you want to do before doing them?	Thinking about which seeds to plant next season; planning the next trip to the local town.	Examples should reflect locally relevant activities that involve planning, distinguishing enjoyment of planning itself from the activity itself.

#	Instrument	Subscale	# in original scale	Question	Translation equivalent in English	Useful examples	Comments
23	AQ	Attention switching	46	New situations make me anxious	Do you feel nervous when you face new situations?	Visiting a new community; going to a new hunting spot.	Examples should emphasize locally relevant "new" contexts that some describe with discomfort. These should be probed and refined during focus groups.
24	AQ	Social skill	48	I am a good diplomat	Do you think you are good at helping people when they have conflicts with each other?	--	--
25	AQ	Attention to detail	49	I am not very good at remembering people's date of birth	Are you very good at remembering things?	People's names; their community of origin; their ancestry (e.g., son of/daughter of); people's birth dates.	Examples aim to tap into what is locally perceived as an indicator of good memory. Probe during focus groups
26	AQ	Attention to detail	5	I often notice small sounds when others do not	Can you pay attention to very small sounds that others cannot?	--	--
27	B2	Social Self-Regulation	11	I tend to be honest	Are you honest, not a liar?	--	--
28	B2	Social Self-Regulation	17	I tend to be respectful	Are you respectful?	--	--
29	B2	Social Self-Regulation	19	I tend to be arrogant	Do you look down on people?	--	--
30	B2	Social Self-Regulation	2	I tend to be compassionate	Do you like helping your friends when they feel sad or have problems?	--	--

#	Instrument	Subscale	# in original scale	Question	Translation equivalent in English	Useful examples	Comments
31	B2	Social Self-Regulation	24	I tend to be hot-tempered	Do you get angry fast?	--	--
32	B2	Dynamism	26	I tend to be bold	Are you brave?	--	--
33	B2	Dynamism	29	I tend to be clever	Are you clever?	--	--
34	B2	Dynamism	30	I tend to be confident	Are you confident or self-assured?	Thinking you can win a match; that you can catch a lot of fish.	Examples were probed during focus groups
35	B2	Dynamism	36	I tend to be happy	Are you happy?	--	--
36	B2	Dynamism	37	I tend to be lively	Do you think you are lively, full of energy, or eager to do things?	--	--
37	B2	Dynamism	40	I tend to be pessimistic	Are you someone who is not trusting of things going well?	When someone goes hunting and you say they will return empty-handed.	Examples were probed during focus groups
38	B2	Dynamism	42	I tend to be sad	Are you sad?	--	--
39	B2	Dynamism	43	I tend to be shy	Are you shy?	--	--
40	B2	Social Self-Regulation	6	I tend to be friendly	Are you friendly?	--	--
41	B2	Social Self-Regulation	7	I tend to be generous	Are you generous?	Sharing food (meat after hunting).	--
42	BFI	Openness	12	I see myself as someone who prefers jobs that are routine	Do you enjoy work that is always the same?	Harvesting rice; weaving a basket or purse.	Examples should focus on tasks that are repetitive and ongoing, rather than one-time activities (e.g., building a house, hunting, fishing).

#	Instrument	Subscale	# in original scale	Question	Translation equivalent in English	Useful examples	Comments
43	BFI	Sociability	13	I see myself as someone who starts disputes with others	Do you start problems or conflicts?	--	--
44	BFI	Conscientiousness	14	I see myself as someone who is a reliable worker, trustworthy.	Do you think you are a reliable and trustworthy worker?	--	--
45	BFI	Extraversion	16	I see myself as someone who tends to be quiet	Are you someone quiet?	--	--
46	BFI	Opennes	17	I see myself as someone who values the artistic, the aesthetic	Do you like things or activities related to dancing, singing, or drawing?	--	Instead of abstract terms such as artistic or aesthetic, the question should refer to concrete activities. It should also be clear that people do not need to perform these activities themselves, they may simply witness them and still enjoy them.
47	BFI	Conscientiousness	18	I see myself as someone who tends to be disorganized	Are you someone who does not prepare?	Not being ready when you need to visit someone or go hunting; not bringing clothes or materials needed for work; being late.	The phrasing should be checked to avoid sounding overly judgmental.
48	BFI	Sociability	24	I see myself as someone who is generally trusting	Do you trust other people?	--	--
49	BFI	Neuroticism	26	I see myself as someone who cares a lot about things	Do you worry a lot about things?	--	--

#	Instrument	Subscale	# in original scale	Question	Translation equivalent in English	Useful examples	Comments
50	BFI	Sociability	28	I see myself as someone who is forgiving, it is not difficult for me to forgive	Is it not hard for you to forgive when someone makes a mistake or bothers you?	--	--
51	BFI	Conscientiousness	29	I see myself as someone who does things efficiently	Do you do things well and fast?	Making a purse; working in a field; building a canoe.	Examples should reflect local activities where both the quality and speed of completion of the task are recognized.
52	BFI	Conscientiousness	3	I see myself as someone who is meticulous at work	Are you detailed and thorough when you work on something?	Pulling up rice; making a "square" field; clearing the field	Examples should capture activities that could be done either "sloppily" or very meticulously
53	BFI	Neuroticism	30	I see myself as someone who is temperamental, moody	Do your ideas and moods change frequently?	--	--
54	BFI	Agreeableness	33	I see myself as someone who is sometimes cold and distant	Are you distant from other people?	--	--
55	BFI	Agreeableness	37	I see myself as someone who is considerate and kind to almost everyone	Are you kind to other people?	--	--
56	BFI	Neuroticism	38	I see myself as someone who gets nervous easily	Do you get nervous easily?	--	--
57	BFI	Extraversion	40	I see myself as someone who is assertive, not afraid to express what I want	Do you know what you like?	Knowing what you want to eat; knowing where you want to go.	Find examples that indicate that the person does not have difficulties making choices.

#	Instrument	Subscale	# in original scale	Question	Translation equivalent in English	Useful examples	Comments
58	BFI	Conscientiousness	42	I see myself as someone who is easily distracted	Do you get distracted easily when you're doing something?	--	--
59	BFI	Sociability	43	I see myself as someone who is outgoing	Do you think you are not shy when talking to people?	--	There is no direct translation for “extroverted”. Interviewers should clarify the intent as being comfortable and open in conversation, not gossiping.
60	BFI	Extraversion	6	I see myself as someone who is reserved	Do you talk about yourself or your problems with other people?	--	Talking with relatives should be included
61	BAPQ	Pragmatic Language	10	My voice has a flat or monotone sound to it.	When you speak, does your voice sound the same, without emotion or enthusiasm?	--	Interviewers often imitate a flat or monotone voice to help respondents understand the intended meaning.
62	BAPQ	Pragmatic Language	14	People ask me to repeat things I’ve said because they don’t understand.	Do people ask you to repeat what you’ve said because they didn’t understand you?	--	Interviewers should make sure this is not due to background noise or the speaker being too loud, but rather about clarity of speech
63	BAPQ	Pragmatic Language	17	I have been told that I talk too much about certain topics.	Have people told you that you talk too much about certain topics?	--	--
64	BAPQ	Rigid	19	I look forward to trying new things.	Do you like trying new things?	New approaches; new flavors; new places to visit	Examples should be adapted to locally relevant experiences invoking novelty

#	Instrument	Subscale	# in original scale	Question	Translation equivalent in English	Useful examples	Comments
65	BAPQ	Rigid	3	I am comfortable with unexpected changes in plans.	Do you get upset when plans change at the last minute?	Being told you would go to the field, to hunt, or to travel, but then the person decides to do something else (e.g., play or not go).	Examples should reflect locally meaningful activities where last-minute changes are common.
66	BAPQ	Aloof	31	I prefer to be alone rather than with others.	Do you like being alone?	Not liking to visit or be visited (sobaqui).	--
67	BAPQ	Rigid	8	I have to warm myself up to the idea of visiting an unfamiliar place.	Do you feel nervous before visiting a place you don't know?	--	--
68	BAPQ	Rigid	26	People get frustrated by my unwillingness to bend.	Do other people get unhappy/unsatisfied because you don't change your way of thinking?	Disagreements about hunting or fishing spots; disagreements about ways of working (e.g., making a recipe, building a house); Marriages	Examples should reflect situations where disagreements are common in daily life.
69	CATI	Cognitive rigidity	14	I like my belongings to be sorted in certain ways and will spend time making sure they are that way	Do you like your things to be organized in a specific way and spend time making sure they are that way?	Clothes; gardens; kitchen items; tools	Examples should reflect areas of daily life where the degree of tidiness varies.
70	CATI	Communication	19	I can tell how people feel from their facial expressions	Can you tell how people feel by looking at their faces?	--	--
71	CATI	Cognitive rigidity	21	I feel discomfort when prevented from completing a particular routine	Do you feel good when you can do the same activities every day?	Waking up around the same time; eat the same as the day before; head to the field; and work with the same crop	Identify meaningful sequence of everyday activities

#	Instrument	Subscale	# in original scale	Question	Translation equivalent in English	Useful examples	Comments
72	CATI	Sensory sensitivity	24	I am over-sensitive to particular tastes (e.g., salty, sour, spicy, or sweet)	Do some tastes feel 'too strong' to you (like salty, sour, spicy, or sweet)?	Salty; sour; spicy; sweet.	In comparison to others who do not seem bothered by the same foods.
73	CATI	Cognitive rigidity	27	I often insist on doing things in a certain way, or re-doing things until they are ‘just right’	Do you keep doing things until they feel right?	Redoing a purse while weaving; remaking a bench or table if it does not come out well; replanting.	Examples should highlight activities where mistakes can lead to starting over.
74	CATI	Repetitive behaviour	32	There are certain repetitive actions that others consider to be ’characteristic’ of me (e.g. stroking my hair)	Do you do repetitive actions that others notice?	Touching your hair; rocking in a chair; playing with objects in your hands; biting or cutting your nails; scratching; getting up and down from your seat or the ground; pulling out grass; cleaning your nails; playing with wool.	Examples should come from focus groups
75	CATI	Sensory sensitivity	36	I react poorly to unexpected loud noises	Do you get scared, hide, or get angry when there are loud and unexpected noises?	Thunder	--
76	CATI	Cognitive rigidity	5	There are certain activities that I always choose to do the same way, every time	Do you choose to do certain activities in the same way?	Making a field; weeding rice	Examples should reflect tasks where people may either repeat the same approach or vary their techniques.

#	Instrument	Subscale	# in original scale	Question	Translation equivalent in English	Useful examples	Comments
77	CATI	Social camouflage	6	Sometimes I watch people interacting and try to copy them when I need to socialise	Do you imitate other people in order to socialize?	Watching how a leader speaks to a group; copying how others joke; joining groups of gossipers or players; friends laughing, playing soccer, or drinking chicha together	Examples should be adapted to reflect everyday social situations.
78	DS-Tsi	--	11	[item from previously adapted scale Tsimane]	In the past month, have you had problems sleeping?	Sleeping more or less than usual; difficulty getting out of bed; waking up several times at night.	Item should capture perceived changes in sleep behavior.
79	DS-Tsi	--	12	[item from previously adapted scale Tsimane]	In the past month, have you perceived changes in your eating?	Loss of appetite; eating more than usual; eating less than usual	Item should focus on noticeable shifts in appetite or food intake.
80	DS-Tsi	--	2	[item from previously adapted scale Tsimane]	In the past month, have you cried more than usual?	--	Item should capture perceived changes in crying frequency or intensity.
81	DS-Tsi	--	4	[item from previously adapted scale Tsimane]	In the past month, have you thought of hurting or killing yourself?	--	--
82	DS-Tsi	--	5	[item from previously adapted scale Tsimane]	In the past month, did you feel like you were a worthless person?	Feeling incapable of being a good parent/offspring, or community member; feeling that you do things worse than before.	Examples should capture feelings of low self-worth in relation to socially valued roles and responsibilities
83	IMHA	Anger Issues	2	I argued with someone at work, at school, or at church.	In the past month, did you have an argument with someone?	At home; at work; at school; at church.	--
84	IMHA	Anxiety	1	I felt afraid.	In the past month, did you feel afraid?	--	--

#	Instrument	Subscale	# in original scale	Question	Translation equivalent in English	Useful examples	Comments
85	IMHA	Anxiety	6	My worrying got in the way of doing something I intended to do.	In the past month, did your worries stop you from doing work that you had to do?"	--	--
86	IMHA	Anxiety	7	I felt shaky or trembly.	In the past month, did you experience tremors out of fear?	--	Interviewers should clarify that the question refers to trembling due to fear, not illness.
87	IMHA	Life stress	1	Stressful things (family illness, financial set backs, accidents) occurred in my life.	In the past month, did something very problematic happen to you?	A family member's illness or death; money problems; a flood; a fire.	Examples of major adverse life events should come from focus groups
88	IMHA	PSTD	1	I had an upsetting dream.	In the past month, did you have a nightmare?	--	--
89	IMHA	Relationship	3	I felt afraid of my romantic partner.	In the past month, did you feel afraid of being with your partner?	--	--
90	IMHA	Relationship	5	My partner physically hurt me.	In the past month, did your partner hit you?	--	--
91	IMHA	Substance Abuse	1	I thought I should cut down on my drinking or drug use.	In the past month, did you think you should reduce your use of alcohol or drugs?	--	--
92	IMHA	Substance Abuse	6	I failed to do what was normally expected from me because of drinking or drug use.	In the past month, did you not do what you were supposed to because of using alcohol or drugs?	--	--

#	Instrument	Subscale	# in original scale	Question	Translation equivalent in English	Useful examples	Comments
93	OCI-R	--	11	How much has sometimes having to wash or clean yourself simply because you feel contaminated distressed or bothered you during the past month?	In the past month, did you get bothered or upset by wanting to clean yourself because you felt "very dirty?	--	--
94	OCI-R	--	13	How much has avoiding throwing things away because you are afraid you might need them later distressed or bothered you during the past month?	In the past month, did you get bothered or upset by not throwing things that you think you could use later?	Motorcycle parts; shoes; roofing sheets; clothes.	Examples should emphasize household items whose future use is not guaranteed, yet are still kept.
95	OCI-R	--	15	How much do you need things to be arranged in a particular order, and how much has this distressed or bothered you during the past month?	In the past month, did you get bothered or upset by things not arranged/organized in the way you like them?	Clothes; shoes; tools around the house.	Very few people express problems with disorganization, but some prefer things to be ordered in specific ways. This variation should be probed during focus groups.
96	OCI-R	--	18	How much has frequently getting nasty thoughts and having difficulty in getting rid of them distressed or bothered you during the past month?	In the past month, have you been bothered or upset by "bad and strong thoughts"?	--	“Bad thoughts” often refer to ideas about hurting oneself or others and are considered taboo.“Strong thoughts” are those that are hard to get rid of and may prevent sleep.

#	Instrument	Subscale	# in original scale	Question	Translation equivalent in English	Useful examples	Comments
97	OCI-R	--	5	How much has finding it difficult to touch an object when you know it has been touched by strangers or certain people distressed or bothered you during the past month?	In the past month, did you get bothered or upset by touching an object that you know has been touched by other people?	A bowl that someone else has already drunk from.	Sharing bowls to drink is a very common practice that usually does not bother people, but some individuals report discomfort. The item should probe for this variation.
98	OCI-R	--	7	How much has collecting things you don't need distressed or bothered you during the past month?	In the past month, did you get bothered or upset by keeping things you don't need?	Old mechanical parts; other unused objects	Examples should come from focus groups to have an idea of what objects can be accumulated. Interviewers should ensure the emphasis is on the respondent feeling bothered or upset by holding on to them
99	SPQ	Suspiciousness	18	Do you often feel that other people have it in for you?	Are there people who don't like you?	--	--
100	SPQ	Excessive social anxiety	2	I sometimes avoid going to places where there will be many people because I will get anxious.	Do you get nervous when you're in places with a lot of people?	--	--
101	SPQ	Excessive social anxiety	20	Do you ever get nervous when someone is walking behind you?	Do you get nervous when someone is walking behind you?	--	--

#	Instrument	Subscale	# in original scale	Question	Translation equivalent in English	Useful examples	Comments
102	SPQ	Ideas of reference	28	Have you ever noticed a common event or object that seemed to be a special sign for you?	Do objects or places have a special sign for you?	Specific birds chanting; the shape of a path, tree, or rock that signals danger	Examples should be refined through focus groups, drawing on situations where things are interpreted as carrying a warning or a “permission/greenlight” for some people but not for others.
103	SPQ	Excessive social anxiety	29	I get anxious when meeting people for the first time.	Do you get nervous when you meet someone for the first time?	--	--
104	SPQ	Odd Beliefs or Magical Thinking	3	Have you had experiences with the supernatural?	Do things related to witchcraft or spirits "come talk" to you?	Talking with spirits; talking to animals, or having animals talk to you; seeing things that others cannot see.	Interviewers should clarify that it refers to personal experiences, not stories heard from others.
105	SPQ	Odd Speech	34	I often ramble on too much when speaking.	When you speak, do you go off topic?	--	Interviewers might "act" as an example of going off topic
106	SPQ	Unusual Perceptual Experiences	40	Have you ever seen things invisible to other people?	Do you see things that others can't see?	--	It should be made clear that the item is not about visual ability.
107	SPQ	Unusual Perceptual Experiences	48	Do everyday things seem unusually large or small?	Do you see things appear larger or smaller than normal?	--	It is important to clarify whether the respondent was alone and others could not see the changes in shape, but only the person themselves.

#	Instrument	Subscale	# in original scale	Question	Translation equivalent in English	Useful examples	Comments
108	SPQ	Unusual Perceptual Experiences	4	Have you often mistaken objects or shadows for people, or noises for voices?	Do you hear sounds like voices that others cannot hear?	--	This item was split to distinguish between auditory and visual perceptions to improve comprehension. The auditory version was retained as visual perception is already asked in another question. It should be made clear that the item is not about hearing ability.
109	SPQ	Constricted affect	51	I tend to avoid eye contact when conversing with others.	Do you look people in the eye when you talk to them?	--	--
110	SPQ	Ideas of reference	53	When you see people talk-ing to each other, do you often wonder if they are talking about you?	When you see others talking, do you think they are talking about you?	--	--
111	SPQ	Odd Beliefs or Magical Thinking	55	Have you ever felt that you are communicating with another person telepathically (by mind-reading)?	Are you able to see what is in someone else's mind?	Like how the shaman does	Clarify whether it is interpreted metaphorically (empathy) or literally (special abilities, "shamanic practices").
112	SPQ	Unusual Perceptual Experiences	56	Does your sense of smell sometimes become unusually strong?	Can your nose smell things very well that others can't smell?	--	--

#	Instrument	Subscale	# in original scale	Question	Translation equivalent in English	Useful examples	Comments
113	SPQ	Suspiciousness	65	Do you often have to keep an eye out to stop people from taking advantage of you?	Do you think you have to protect yourself so that others don't take advantage of you?	When someone asks to borrow something; when selling a product in town; when asked to contribute in a community project, when working for wages.	Examples should come from focus groups after discussions of situations where some people may feel vulnerable to exploitation and others do not.
114	SPQ	Suspiciousness	9	I am sure I am being talked about behind my back.	Do others talk about you behind your back?	--	--
115	WS	Magical ideation	5	At times, I perform certain little rituals to ward off negative influences.	Do you perform rituals or traditions to ward off evil spirits or bad luck?	Specific birdsong; wearing a protective necklace or bracelet	There are many protective traditions, and they can vary slightly by region. Local examples should be solicited during interviews or focus groups to ensure cultural relevance.
116	WS	Perceptual aberration	6	Sometimes I have had feelings that I am united with an object near me.	Do you feel that you are connected to an object near you?	A special stone; objects used by shamans such as stones or dolls; amulets worn as necklaces or bracelets.	Traditional healers often describe very strong ties to specific objects, and mentioning their behavior can quickly convey the intended meaning. Some objects are believed to bring good fortune and, if lost or broken, are seen as a bad omen. For some people, these objects are deeply connected to the self, while others report no such connection.

#	Instrument	Subscale	# in original scale	Question	Translation equivalent in English	Useful examples	Comments
117	WS	Social anhedonia	4	Just being with friends can make me feel really good	Do you like being with your friends?	Being at a chicha party; playing soccer; visiting or being visited	It is important to nudge respondents toward moments when they would be with “friends.” These may include relatives, since many people describe not having friends but kin.

Table SM8. Complete list of "original" items abandoned for cross-cultural limitations

Abbreviations: ASRS = Adult ADHD Self-Report; AQ = Autism Quotient; B2 = Cross-Cultural Big Two; BAPQ = Broad Autism Phenotype Questionnaire; BFI = Big Five Inventory; CATI = Comprehensive Autistic Trait Inventory; DS-Tsi = Depressive Symptoms (Tsimane'); GAD = Generalized Anxiety Disorder; IMHA = International Mental Health Assessment; OCI-R = Obsessive-Compulsive Inventory–Revised; SPQ = Schizotypal Personality Questionnaire; WS = Wisconsin Scales of Vulnerability to Psychosis–Brief.

Instrument	Subscale	Question	Brief Explanations
ASRS	Hyperactivity-Impulsivity	14. How often do you have difficulty unwinding and relaxing when you have time to yourself?	We found it difficult to convey the idea of relaxing or having time off. The notion of taking a break from responsibilities did not translate well and lacked a culturally meaningful equivalent.
ASRS	Inattention	2. How often do you have difficulty getting things in order when you have to do a task that requires organisation?	We found it difficult to convey the idea of getting things in order for a task that requires organization. Participants often interpreted “getting things in order” as physical activity (cleaning). Comprehension improved when we used concrete examples such as planning a visit or a hunting trip (deciding the route, bringing tools/food, setting a time to leave). Also, external factors were mentioned, such as lack of money or fatigue, as factors impeding the realization of the action. We explicitly tried to distinguish this construct from cleanliness and from resource scarcity, but ultimately failed.
ASRS	Inattention	4. When you have a task that requires a lot of thought, how often do you avoid or delay getting started?	Participants understood “a task that requires a lot of thought” as complex activities, such as building a house or canoe, or planning a pattern in a purse. However, the idea of delaying getting started was not easily understood, as often starting a project is contingent on other factors; instead, participants more readily understood starting a task, but not finishing it already encompassed in another item.

Instrument	Subscale	Question	Brief Explanations
AQ	Attention switching	10 I can easily keep track of several different people's conversations	We found it difficult to convey the idea of keeping track of several conversations. Participants often interpreted the item as failing to follow a conversation due to being tired.
AQ	Attention to detail	19 I am fascinated by numbers	Phone numbers, or numbers in general, were not meaningful in the local context, and no suitable equivalents could be identified.
AQ	Attention to detail	23 I notice patterns in things all the time	The concept of "noticing patterns" was not understood without additional context. Participants required concrete examples (e.g., recognizing repeated forms in nature or objects) to make sense of the item. No meaningful and general example was found
AQ	Attention to detail	29 I am not very good at remembering phone numbers	Phone numbers, or numbers in general, were not meaningful in the local context, and no suitable equivalents could be identified.
AQ	Attention to detail	9 I am fascinated by dates	Dates are not necessarily meaningful in the local context; no suitable equivalents could be identified.
AQ	Communication	33 When I talk on the phone, I am not sure when it's my turn to speak	We found it difficult to convey the idea of "awareness of turn-taking" in conversation. Alternative phrasings that asked more explicitly whether a person interrupts others, or that focused on the person's general talkativeness, were easier to understand. Phones are not appropriate examples in this context.

Instrument	Subscale	Question	Brief Explanations
AQ	Communication	18 When I talk, it isn't always easy for others to get a word in edgeways	We found it difficult to convey the idea of "awareness of turn-taking" in conversation. Alternative phrasings that asked more explicitly whether a person interrupts others, or that focused on the person's general talkativeness, were easier to understand. Phones are not appropriate examples in this context.
AQ	Communication	26 I don't know how to keep a conversation going	We found it difficult to convey the idea of "awareness of turn-taking" in conversation. Alternative phrasings that asked more explicitly whether a person interrupts others, or that focused on the person's general talkativeness, were easier to understand. Phones are not appropriate examples in this context.
AQ	Communication	27 I find it easy to ''read between the lines''	We found it difficult to convey the idea of "reading between the lines." Asking whether the person can understand a joke was more concrete and served as a clearer example of the more complex use of pragmatic language.
AQ	Communication	7 Other people frequently tell me that what I have said is impolite	The concepts of politeness and respect were intrinsically confounded in this context. For this reason, items referring specifically to politeness were abandoned.
AQ	Imagination	24 I would rather go to the theatre than a museum	Museums and theatres are not meaningful in this context. The underlying construct of whether a person enjoys activities that involve others is already encompassed in another item.

Instrument	Subscale	Question	Brief Explanations
AQ	Imagination	42 I find it difficult to imagine what it would be like to be someone else	We attempted to make the item more concrete by giving examples, such as imagining being the president or belonging to another family or community. However, even with these prompts, the item was most often not understood.
AQ	Imagination	20 Reading a story, I find it difficult to work out the characters' intentions	The item "work out the characters' intentions" was too abstract, as was the request to "imagine something" without further context.
AQ	Imagination	3 Trying to imagine something, I find it easy to create a picture in my mind	The item "work out the characters' intentions" was too abstract, as was the request to "imagine something" without further context.
AQ	Imagination	8 Reading a story, I can easily imagine what the characters might look like	Because of low literacy, asking participants to imagine characters through reading was not appropriate. Instead, we adapted the question to refer to hearing stories through storytelling. However, participants tended to respond to whether they enjoyed listening to stories, rather than addressing whether they imagined the characters. When asked why they sometimes did not enjoy stories, some explained that it was because imagining the characters or the story was too difficult. Given this, we dropped this item and instead asked directly whether participants enjoyed listening to stories or not in another item.

Instrument	Subscale	Question	Brief Explanations
AQ	Social skill	22 I find it hard to make new friends	Participants did not tend to distinguish between making new friends and meeting new people. This posed a problem, as friendships often occur between kin, whom participants already knew, even if they might become closer later. To avoid confusion, this item was dropped.
AQ	Social skill	45 I find it difficult to work out people's intentions	We found it difficult to convey the idea of taking someone else's intentions. Participants more easily understood questions framed from their own perspective and only secondarily from another person's. This construct was therefore better captured by other questions.
BFI	Agreeableness	41. I see myself as someone who likes to cooperate with others	The item "likes to cooperate" was initially translated as "likes working with other people." Participants generally understood this to mean that they preferred working with others because work is usually better or more successful when it involves more people. Other interpretations included enjoying being with others or being generous, constructs that were already encompassed by other items.
BFI	Openness	39. I see myself as someone who is educated in art, music, or literature	Being educated in art, music, or literature is not an appropriate question for this context, as they are very linked to literacy

Instrument	Subscale	Question	Brief Explanations
BFI	Openness	10I see myself as someone who has very diverse interests	The item “diverse interests” was too vague and difficult to translate without examples. When examples were provided (e.g., enjoying both playing football and going to church), participants tended to focus narrowly on those specific activities rather than on the broader construct. For this reason, the item was dropped.
BAPQ	Aloof	18. When I make conversation, it is just to be polite.	The concepts of politeness and respect were intrinsically confounded in this context. For this reason, items referring specifically to politeness were abandoned.
BAPQ	Aloof	23. I am good at making small talk.	Social chitchat was not meaningful in the local context. While formulaic greetings do exist, they are understood as expressions of respect or politeness rather than casual conversation.
BAPQ	Aloof	25. I feel like I am really connecting with other people.	The concepts of “feeling connected,” “disconnected,” “out of sync,” or “in tune with other people” were too abstract and often led to confusion.
BAPQ	Aloof	5. I would rather talk to people to get information than to socialize.	The distinction between the two goals of talking to someone was too abstract and often led to confusion.

Instrument	Subscale	Question	Brief Explanations
BAPQ	Pragmatic Language	20a. I speak too loudly or softly.	This item was initially split into two separate questions—“Do you speak too loudly?” and “Do you speak too softly?”, to improve comprehension. However, participants often became confused, interpreting the items as referring to situations where one might need to yell or whisper, or to cases where the interlocutor had hearing problems and required an adjustment in speech. Since another item already encompassed awareness of voice tone, we retained that one and excluded these.
BAPQ	Pragmatic Language	20b. I speak too loudly or softly.	This item was initially split into two separate questions—“Do you speak too loudly?” and “Do you speak too softly?”—to improve comprehension. However, participants often became confused, interpreting the items as referring to situations where one might need to yell or whisper, or to cases where the interlocutor had hearing problems and required an adjustment in speech. Since another item already encompassed awareness of voice tone, we retained that one and excluded these.
BAPQ	Pragmatic Language	34. I can tell when it is time to change topics in conversation.	We found it difficult to convey the idea of “awareness of topic” in conversation. Alternative phrasings that asked more explicitly whether other people told the participant that they talk too much about certain topics, or whether they tend to go off topic, were easier to understand.

Instrument	Subscale	Question	Brief Explanations
BAPQ	Pragmatic Language	11. I feel disconnected or ‘‘out of sync’’ in conversations with others.	The concepts of “feeling connected,” “disconnected,” “out of sync,” or “in tune with other people” were too abstract and often led to confusion.
BAPQ	Pragmatic Language	7. I am ‘‘in-tune’’ with the other person during conversation.	The concepts of “feeling connected,” “disconnected,” “out of sync,” or “in tune with other people” were too abstract and often led to confusion.
BAPQ	Pragmatic Language	4. It’s hard for me to avoid getting sidetracked in conversation.	We found it difficult to convey the idea of avoiding getting sidetracked in conversation. Participants often interpreted the item as forgetting what they were saying, being tired, not knowing the answer, or not understanding. Comprehension improved slightly with concrete examples already encompassed by other items.
CATI	Communication	33. Metaphors or ‘figures of speech’ often confuse me	There was no adequate word equivalent for figures of speech or metaphors. Providing examples only increased confusion among participants. Understanding jokes served as a clearer and more culturally meaningful example of complex pragmatic language.
CATI	Communication	37. I have difficulty understanding someone else’s point of view	We found it difficult to convey the idea of taking someone else’s point of view. Participants more easily understood questions framed from their own perspective and only secondarily from another person’s. This construct was therefore better captured by other questions.

Instrument	Subscale	Question	Brief Explanations
CATI	Repetitive behaviour	25. I engage in certain repetitive actions when I feel stressed	The association between feeling stressed—a concept without a perfect translation—and engaging in repetitive actions did not necessarily exist in a conscious manner. For example, when asked about behaviors such as nail-biting or pulling grass from the ground, participants could recognize the repetitive behavior itself but did not link it to feeling distressed. As a result, the requirement that respondents explicitly associate repetitive behaviors with being worried or distressed was dropped.
CATI	Repetitive behaviour	12. There are certain objects that I fiddle or play with that can help me calm down or collect my thoughts	The association between feeling stressed—a concept without a perfect translation—and engaging in repetitive actions did not necessarily exist in a conscious manner. For example, when asked about behaviors such as nail-biting or pulling grass from the ground, participants could recognize the repetitive behavior itself but did not link it to feeling distressed. As a result, the requirement that respondents explicitly associate repetitive behaviors with being worried or distressed was dropped.
CATI	Sensory sensitivity	31. Sometimes the presence of a smell makes it hard for me to focus on anything else	Participants often misunderstood the question, interpreting it subjectively in terms of what constitutes a good or bad smell, rather than in relation to whether a smell would interrupt what they were doing.

Instrument	Subscale	Question	Brief Explanations
CATI	Sensory sensitivity	4. I am over-sensitive to bright lighting I would describe myself as a very sensory-sensitive person	Examples used to assess the degree of sensitivity to light showed no variation, as nearly all participants reported being very bothered by flickering lights or by bright light (such as sunlight reflected in a mirror). Some interesting examples did emerge, such as people who could not sleep with nearby lights on; however, because access to artificial light is not widespread in this context, the relevance of such items was limited.
CATI	Sensory sensitivity	40. I am sensitive to flickering lights	Examples used to assess the degree of sensitivity to light showed no variation, as nearly all participants reported being very bothered by flickering lights or by bright light (such as sunlight reflected in a mirror). Some interesting examples did emerge, such as people who could not sleep with nearby lights on; however, because access to artificial light is not widespread in this context, the relevance of such items was limited.

Instrument	Subscale	Question	Brief Explanations
CATI	Sensory sensitivity	11. There are times when I feel that my senses are overloaded	We found it difficult to explain the concept of sensory overload in general terms. Participants understood it better when we used modality-specific, concrete examples, e.g., very loud environments (music, shouting, crowded buses/markets), bright or flickering lights (bright sun, artificial light), strong smells (smoke, fuel, cooking), or uncomfortable touch (tight/itchy clothing, jostling in crowds). All encompassed in other questions already. Emphasizing that overload can occur when several of these happen at once helped, but we needed to go over all the other examples first. People often confuse the idea with being uncomfortable or illness; framing questions around specific triggers and relief after leaving the environment improved comprehension.
CATI	Social camouflage	16. When interacting with other people, I spend a lot of effort monitoring how I am coming across	The concept of “monitoring how I’m coming across” was very difficult to convey and often led to confusion.
CATI	Social camouflage	3. I expend a lot of mental energy trying to fit in with others	The concept of mental energy was often conflated with simply feeling tired, being sick, or being shy, rather than referring to cognitive or emotional effort.
CATI	Social camouflage	9. I look for strategies and ways to appear more sociable	The concept of social strategies required more concrete examples for participants to understand, and was already encompassed by other items.

Instrument	Subscale	Question	Brief Explanations
CATI	Social interactions	35. I find it difficult to make new friends	Participants did not tend to distinguish between making new friends and meeting new people. This posed a problem, as friendships often occur between kin, whom participants already knew, even if they might become closer later. To avoid confusion, this item was dropped.
B2	Social Self-Regulation	23. inhumane	The concept of inhumanity was often conflated with cruelty. Research assistants felt that participants would be unlikely to answer such questions honestly. Instead, it was considered more appropriate to ask about goodness
GAD	--	4. Trouble relaxing	We found it difficult to convey the idea of relaxing or having time off. The notion of taking a break from responsibilities did not translate well and lacked a culturally meaningful equivalent.

Instrument	Subscale	Question	Brief Explanations
IMHA	ADHD	IMHA_ADHD1.I had difficulty getting things in order when I had to do a task that required organisation.	We found it difficult to convey the idea of getting things in order for a task that requires organization. Participants often interpreted “getting things in order” as physical activity (cleaning). Comprehension improved when we used concrete examples such as planning a visit or a hunting trip (deciding the route, bringing tools/food, setting a time to leave). Also, external factors were mentioned, such as lack of money or fatigue, as factors impeding the realization of the action. We explicitly tried to distinguish this construct from cleanliness and from resource scarcity, but ultimately failed.
IMHA	ADHD	IMHA_ADHD5.When I had a task that required a lot of thought, I avoided or delayed getting started.	Participants understood “a task that requires a lot of thought” as complex activities, such as building a house or canoe, or planning a pattern in a purse. However, the idea of delaying getting started was not easily understood, as often starting a project is contingent on other factors; instead, participants more readily understood starting a task, but not finishing it already encompassed in another item.
IMHA	Anxiety	IMHA_anxiety5.My heart beat fast (not due to exercise).	The item “My heart beats fast (not due to exercise)” was problematic. Even when physical activity was explicitly removed as an explanation, participants still did not understand the distinction and often appeared confused.

Instrument	Subscale	Question	Brief Explanations
IMHA	Anxiety	IMHA_anxiety2.I got dizzy or lightheaded.	It was difficult to disentangle the experience from other common causes such as dehydration, excessive sun exposure, fatigue, or standing up too quickly.
IMHA	Anxiety	IMHA_anxiety4.I avoided a situation that made me nervous or anxious.	We found it difficult to convey the idea of avoiding a situation without providing very concrete examples. Situations such as visiting a new place were more appropriate and easier for participants to understand.
IMHA	Life stress	IMHA_life_stress2.There was more I needed to do than could fit into the day.	The item "There was more I needed to do than could fit into the day" was not meaningful, as participants considered this to always be true.
IMHA	Life stress	IMHA_life_stress4.I had so many responsibilities that there was no time to relax.	We found it difficult to convey the idea of relaxing or having time off. The notion of taking a break from responsibilities did not translate well and lacked a culturally meaningful equivalent.
IMHA	PTSD	IMHA_PTS4.I avoided an activity or situation that reminded me of a stressful experience from the past.	We found it difficult to convey the idea of stressful past situations or experiences without very concrete examples. Illustrations such as being in a serious accident or witnessing one (e.g., a bridge collapse or a car/motorcycle accident) were somewhat helpful, but still did not allow participants to connect easily to their own traumatic experiences. In contrast, asking about nightmares proved more systematic and meaningful, as people were able to relate to this more readily.

Instrument	Subscale	Question	Brief Explanations
IMHA	PTSD	IMHA_PTS5.I had physical reactions (heart pounding, trouble breathing, sweating) when something reminded me of a stressful experience from the past.	We found it difficult to convey the idea of stressful past situations or experiences without very concrete examples. Illustrations such as being in a serious accident or witnessing one (e.g., a bridge collapse or a car/motorcycle accident) were somewhat helpful, but still did not allow participants to connect easily to their own traumatic experiences. In contrast, asking about nightmares proved more systematic and meaningful, as people were able to relate to this more readily.
IMHA	PTSD	IMHA_PTS6.I suddenly felt or acted as if a stressful experience were happening again (as if I were reliving it).	We found it difficult to convey the idea of stressful past situations or experiences without very concrete examples. Illustrations such as being in a serious accident or witnessing one (e.g., a bridge collapse or a car/motorcycle accident) were somewhat helpful, but still did not allow participants to connect easily to their own traumatic experiences. In contrast, asking about nightmares proved more systematic and meaningful, as people were able to relate to this more readily.

Instrument	Subscale	Question	Brief Explanations
IMHA	PTSD	IMHA_PTS3.I had disturbing memories, thoughts, or images of a stressful experience from the past.	We found it difficult to convey the idea of stressful past situations or experiences without very concrete examples. Illustrations such as being in a serious accident or witnessing one (e.g., a bridge collapse or a car/motorcycle accident) were somewhat helpful, but still did not allow participants to connect easily to their own traumatic experiences. In contrast, asking about nightmares proved more systematic and meaningful, as people were able to relate to this more readily.
IMHA	Relationship	IMHA_Relationship1.Arguments with my partner went unresolved.	In this context, arguments were not typically described in terms of resolution or lack of resolution, making the construct difficult to understand.
OCI-R	--	10. How much do you feel you have to repeat certain numbers, and how much has this distressed or bothered you during the past month?	The item “feeling you have to repeat certain numbers” or counting was culturally inappropriate. Participants instead mentioned that repeating prayers could sometimes be helpful, which we concluded reflected a different behavior.
OCI-R	--	16. How much do you feel that there are good and bad numbers, and how much has this distressed or bothered you during the past month?	The item “good and bad numbers” was culturally inappropriate.

Instrument	Subscale	Question	Brief Explanations
OCI-R	--	4. How much has feeling compelled to count while you are doing things distressed or bothered you during the past month?	The item “feeling you have to repeat certain numbers” or counting was culturally inappropriate. Participants instead mentioned that repeating prayers could sometimes be helpful, which we concluded reflected a different behavior.
OCI-R	--	14. How much has repeatedly checking gas and water taps and light switches after turning them off distressed or bothered you during the past month?	We found it difficult to convey the idea of “checking things more often than necessary” without providing concrete, meaningful examples. Examples that resonated more included: checking whether a flashlight was turned off, making sure a door was closed (but not everyone had a closing door, or verifying that no food was left behind when visiting someone or going hunting. However, even with these more specific illustrations, participants often did not fully understand the question. Objects such as windows, drawers, gas, or electricity were not meaningful in this context.

Instrument	Subscale	Question	Brief Explanations
OCI-R	--	2. How much has checking things more often than necessary distressed or bothered you during the past month?	We found it difficult to convey the idea of “checking things more often than necessary” without providing concrete, meaningful examples. Examples that resonated more included: checking whether a flashlight was turned off, making sure a door was closed (but not everyone had a closing door, or verifying that no food was left behind when visiting someone or going hunting. However, even with these more specific illustrations, participants often did not fully understand the question. Objects such as windows, drawers, gas or electricity were not meaningful in this context.
OCI-R	--	8. How much has repeatedly checking doors, windows, drawers, etc., distressed or bothered you during the past month?	We found it difficult to convey the idea of “checking things more often than necessary” without providing concrete, meaningful examples. Examples that resonated more included: checking whether a flashlight was turned off, making sure a door was closed (but not everyone had a closing door, or verifying that no food was left behind when visiting someone or going hunting. However, even with these more specific illustrations, participants often did not fully understand the question. Objects such as windows, drawers, gas, or electricity were not meaningful in this context.

Instrument	Subscale	Question	Brief Explanations
SPQ	Constricted affect	43. I am poor at returning social courtesies and gestures.	We found it difficult to convey the concept of “social courtesies and gestures.” A better alternative was asking about looking someone in the eyes, which was more concrete and meaningful for participants.
SPQ	Excessive social anxiety	11. I get very nervous when I have to make polite conversation.	The concepts of politeness and respect were intrinsically confounded in this context. For this reason, items referring specifically to politeness were abandoned.
SPQ	Excessive social anxiety	54. I would feel very anxious if I had to give a speech in front of a large group of people.	Public speaking in front of large groups is not a common experience.
SPQ	Ideas of reference	60. Do you sometimes feel that other people are watching you?	We encountered difficulties in conveying items related to drawing attention. For example, “other people are watching you” was often misunderstood. Participants tended to interpret it in concrete situations, such as wearing something unusual (like a disguise) or, for women, when a man was “checking them out.”
SPQ	Ideas of reference	10. I am aware that people notice me when I go out for a meal or to see a film.	We encountered difficulties in conveying items related to drawing attention. For example, “other people are watching you” was often misunderstood. Participants tended to interpret it in concrete situations, such as wearing something unusual (like a disguise) or, for women, when a man was “checking them out.”

Instrument	Subscale	Question	Brief Explanations
SPQ	Ideas of reference	45. When shopping, do you get the feeling that other people are taking notice of you?	We encountered difficulties in conveying items related to drawing attention. For example, “other people are watching you” was often misunderstood. Participants tended to interpret it in concrete situations, such as wearing something unusual (like a disguise) or, for women, when a man was “checking them out.”
SPQ	Ideas of reference	19. Do some people drop hints about you or say things with a double meaning?	Difficulty conveying "drop hints about you or say things with a double meaning". Sometimes it was interpreted as gossip or making jokes at someone’s expense, and other times as hints about a person’s sexuality, a taboo topic that participants were unlikely to respond to honestly.
SPQ	No Closed Friends	41. Do you feel that there is no one you are really close to outside of your immediate family, or people you can confide in or talk to about personal problems?	The notion of confiding in someone outside the family was not culturally appropriate, as personal matters were generally discussed within kin networks.
SPQ	No Closed Friends	49. Writing letters to friends is more trouble than it is worth.	Writing is not a common practice in this context. While text messages were sometimes mentioned, they are not widespread enough to serve as a meaningful example. Simply talking to a friend was a more culturally appropriate alternative.

Instrument	Subscale	Question	Brief Explanations
SPQ	Odd Beliefs or Magical Thinking	39. Can other people feel your feelings when they are not there?	We found it difficult to convey the idea of "feeling your feelings when they are not there." It was more concrete to ask about other sensory experiences, such as seeing or hearing. Participants also tended to interpret the item in terms of shared feelings after an event. For example, both people feel upset at the same time after a fight.
SPQ	Odd Beliefs or Magical Thinking	47. Have you had experiences with astrology, seeing the future, UFOs, ESP, or a sixth sense?	These concepts were either unfamiliar or not meaningful in the local context. Other item already ask more cultural appropriate question
SPQ	Odd or Eccentric Behavior	70. I have some eccentric (odd) habits.	The concept of "eccentricity, oddness, or strangeness was very difficult to translate and consistently led to confusion among participants.
SPQ	Odd or Eccentric Behavior	14. People sometimes comment on my unusual mannerisms and habits.	The concept of "eccentricity, oddness, or strangeness was very difficult to translate and consistently led to confusion among participants.
SPQ	Odd or Eccentric Behavior	23. Sometimes other people think that I am a little strange.	The concept of "eccentricity, oddness, or strangeness was very difficult to translate and consistently led to confusion among participants.
SPQ	Odd or Eccentric Behavior	32. Some people think that I am a very bizarre person.	The concept of "eccentricity, oddness, or strangeness was very difficult to translate and consistently led to confusion among participants.

Instrument	Subscale	Question	Brief Explanations
SPQ	Odd or Eccentric Behavior	5. Other people see me as slightly eccentric (odd).	The concept of “eccentricity, oddness, or strangeness was very difficult to translate and consistently led to confusion among participants.
SPQ	Odd or Eccentric Behavior	67. I am an odd, unusual person.	The concept of “eccentricity, oddness, or strangeness was very difficult to translate and consistently led to confusion among participants.
SPQ	Odd or Eccentric Behavior	74. People sometimes stare at me because of my odd appearance.	The concept of “eccentricity, oddness, or strangeness was very difficult to translate and consistently led to confusion among participants.
SPQ	Odd Speech	42. Some people find me a bit vague and elusive during a conversation.	We found it difficult to convey the idea of “elusiveness or vagueness” in conversation. Alternative phrasings that asked more explicitly whether other people said the participant tends to go off topic or whether others asked them to repeat themselves were easier to understand.
SPQ	Odd Speech	50. I sometimes use words in unusual ways.	The "use of words" in an unusual way was understood by participants as differences in language skills. For example, speaking more eloquently or having a richer vocabulary. These interpretations were strongly linked to education.
SPQ	Suspiciousness	44. Do you often pick up hidden threats or put-downs from what people say or do?	We found it difficult to convey the idea of subtle or implied meanings in what someone else says.

Instrument	Subscale	Question	Brief Explanations
SPQ	Unusual Perceptual Experiences	22. When you look at a person, or yourself in a mirror, have you ever seen the face change right before your eyes?	Some participants understood the question as intended, but others interpreted it in terms of being able to tell whether someone had aged or was becoming sick, which led to confusion.
SPQ	Unusual Perceptual Experiences	31. I often hear a voice speaking my thoughts aloud.	We found it difficult to convey the idea of “hearing a voice speaking one’s thoughts.” The majority of participants interpreted this as their own inner monologue rather than as an external auditory experience.
SPQ	Unusual Perceptual Experiences	64. Are your thoughts sometimes so strong that you can almost hear them?	We found it difficult to convey the idea of “thoughts being strong.” The majority of participants tended to interpret this as overthinking.
WS	Magical ideation	15. I have worried that people on other planets may be influencing what happens on Earth.	The item “I have worried that people on other planets may be influencing what happens on Earth” was adapted to refer instead to “outside forces” in a way that was more culturally meaningful. However, even with this adaptation, the item continued to present significant comprehension issues and was often conflated with religious beliefs.
WS	Magical ideation	4. I have had the momentary feeling that someone’s place has been taken by a look-alike.	The concept of a “look-alike” was often misunderstood. Participants tended to interpret it as being mistaken for someone else (such as a sibling) because of family resemblance.

Instrument	Subscale	Question	Brief Explanations
WS	Magical ideation	9. The hand motions that strangers make seem to influence me at times.	Participants often interpreted "hand motions" literally, as gestures used to communicate (e.g., waving someone over), rather than as an unusual or involuntary influence.
WS	Magical ideation	2. I have occasionally had the silly feeling that a TV or radio broadcaster knew I was listening to him.	Despite participants being familiar with radio, the concept in this item was very difficult to explain and not well understood.
WS	Magical ideation	11. Numbers like 13 and 7 have no special powers.	Referring to specific numbers (or numbers in general) as bearers of luck was not appropriate in this context. Instead, participants referred to specific bird songs as meaningful, but this construct was already encompassed in another item.
WS	Magical ideation	14. Horoscopes are right too often for it to be a coincidence.	These concepts were either unfamiliar or not meaningful in the local context. Other item already ask more cultural appropriate question
WS	Magical ideation	3. I have noticed sounds on my records that are not there at other times.	These concepts were either unfamiliar or not meaningful in the local context. Other item already ask more cultural appropriate question
WS	Magical ideation	7. If reincarnation were true, it would explain some unusual experiences I have had.	These concepts were either unfamiliar or not meaningful in the local context. Other item already ask more cultural appropriate question

Instrument	Subscale	Question	Brief Explanations
WS	Magical ideation	8. I have sometimes had the passing thought that strangers are in love with me.	“Strangers falling in love with me” was not appropriate, as encounters with "true" strangers are uncommon in this context. It was also frequently misunderstood as referring to flirting.
WS	Magical ideation	12. I have had the momentary feeling that I might not be human.	The concept of “not feeling human” was better understood when framed as being a specific type of spirit, which was appropriate in this context. However, this interpretation was very heavily linked to religious beliefs, making the item problematic.
WS	Perceptual aberration	13. Sometimes I feel like everything around me is tilting.	The item “Sometimes I feel like everything around me is tilting” was difficult to convey. Participants often interpreted it concretely in terms of "personal imbalance", such as dizziness from standing up too quickly, fever, dehydration, or fatigue, rather than as a perceptual disturbance.
WS	Perceptual aberration	10. I sometimes have to touch myself to make sure I’m still there.	The item “I sometimes have to touch myself to make sure I’m still there” was difficult to convey. Participants interpret it literally rather than as a "dissociative experience".

Instrument	Subscale	Question	Brief Explanations
WS	Perceptual aberration	7. Sometimes I have had a passing thought that some part of my body was rotting away.	We found it challenging to convey items describing unusual or distorted bodily experiences. Participants tended to interpret these statements in very concrete, literal ways rather than as abstract or metaphorical experiences. For example, “My body was rotting away” was often understood as referring to visible bruises. “My arms or legs are disconnected from the rest of my body” was interpreted as tingling or numbness in the limbs.“My body has become misshapen”, “my body occasionally seems dead or unreal” and “My limb has an unusual shape” were all linked to memories of broken or twisted limbs or physical injury.“My body is decaying inside” was associated with episodes of illness, such as vomiting or diarrhea.

Instrument	Subscale	Question	Brief Explanations
WS	Perceptual aberration	11. I have sometimes had the feeling that one of my arms or legs is disconnected from the rest of my body.	We found it challenging to convey items describing unusual or distorted bodily experiences. Participants tended to interpret these statements in very concrete, literal ways rather than as abstract or metaphorical experiences. For example, “My body was rotting away” was often understood as referring to visible bruises. “My arms or legs are disconnected from the rest of my body” was interpreted as tingling or numbness in the limbs.“My body has become misshapen”, “my body occasionally seems dead or unreal” and “My limb has an unusual shape” were all linked to memories of broken or twisted limbs or physical injury.“My body is decaying inside” was associated with episodes of illness, such as vomiting or diarrhea.

Instrument	Subscale	Question	Brief Explanations
WS	Perceptual aberration	12. I have had the momentary feeling that my body has become misshapen.	We found it challenging to convey items describing unusual or distorted bodily experiences. Participants tended to interpret these statements in very concrete, literal ways rather than as abstract or metaphorical experiences. For example, “My body was rotting away” was often understood as referring to visible bruises. “My arms or legs are disconnected from the rest of my body” was interpreted as tingling or numbness in the limbs.“My body has become misshapen”, “my body occasionally seems dead or unreal” and “My limb has an unusual shape” were all linked to memories of broken or twisted limbs or physical injury.“My body is decaying inside” was associated with episodes of illness, such as vomiting or diarrhea.

Instrument	Subscale	Question	Brief Explanations
WS	Perceptual aberration	14. Parts of my body occasionally seem dead or unreal.	We found it challenging to convey items describing unusual or distorted bodily experiences. Participants tended to interpret these statements in very concrete, literal ways rather than as abstract or metaphorical experiences. For example, “My body was rotting away” was often understood as referring to visible bruises. “My arms or legs are disconnected from the rest of my body” was interpreted as tingling or numbness in the limbs.“My body has become misshapen”, “my body occasionally seems dead or unreal” and “My limb has an unusual shape” were all linked to memories of broken or twisted limbs or physical injury.“My body is decaying inside” was associated with episodes of illness, such as vomiting or diarrhea.

Instrument	Subscale	Question	Brief Explanations
WS	Perceptual aberration	3. I have sometimes had the feeling that my body is decaying inside.	We found it challenging to convey items describing unusual or distorted bodily experiences. Participants tended to interpret these statements in very concrete, literal ways rather than as abstract or metaphorical experiences. For example, “My body was rotting away” was often understood as referring to visible bruises. “My arms or legs are disconnected from the rest of my body” was interpreted as tingling or numbness in the limbs.“My body has become misshapen”, “my body occasionally seems dead or unreal” and “My limb has an unusual shape” were all linked to memories of broken or twisted limbs or physical injury.“My body is decaying inside” was associated with episodes of illness, such as vomiting or diarrhea.

Instrument	Subscale	Question	Brief Explanations
WS	Perceptual aberration	9. I can remember when it seemed as though one of my limbs took on an unusual shape.	We found it challenging to convey items describing unusual or distorted bodily experiences. Participants tended to interpret these statements in very concrete, literal ways rather than as abstract or metaphorical experiences. For example, "My body was rotting away" was often understood as referring to visible bruises. "My arms or legs are disconnected from the rest of my body" was interpreted as tingling or numbness in the limbs."My body has become misshapen", "my body occasionally seems dead or unreal" and "My limb has an unusual shape" were all linked to memories of broken or twisted limbs or physical injury."My body is decaying inside" was associated with episodes of illness, such as vomiting or diarrhea.
WS	Physical anhedonia	13. The first winter snowfall has often looked pretty to me.	Phrases such as "the first winter snowfall" or "a good soap lather when bathing" referred to experiences that do not exist or are not meaningful in this context. Other items, such as "a brisk walk," "watching sunsets," "looking at the stars," "listening to rustling leaves or rain on the roof," or "petting small animals", carried cultural connotations that were not necessarily associated with pleasurable experiences. Overall, the broader category of physical anhedonia failed to provide examples that were universally relevant for participants.

Instrument	Subscale	Question	Brief Explanations
WS	Physical anhedonia	14. A good soap lather when I'm bathing has sometimes soothed and refreshed me.	Phrases such as "the first winter snowfall" or "a good soap lather when bathing" referred to experiences that do not exist or are not meaningful in this context. Other items, such as "a brisk walk," "watching sunsets," "looking at the stars," "listening to rustling leaves or rain on the roof," or "petting small animals", carried cultural connotations that were not necessarily associated with pleasurable experiences. Overall, the broader category of physical anhedonia failed to provide examples that were universally relevant for participants.
WS	Physical anhedonia	2. A brisk walk has sometimes made me feel good all over.	Phrases such as "the first winter snowfall" or "a good soap lather when bathing" referred to experiences that do not exist or are not meaningful in this context. Other items, such as "a brisk walk," "watching sunsets," "looking at the stars," "listening to rustling leaves or rain on the roof," or "petting small animals", carried cultural connotations that were not necessarily associated with pleasurable experiences. Overall, the broader category of physical anhedonia failed to provide examples that were universally relevant for participants.

Instrument	Subscale	Question	Brief Explanations
WS	Physical anhedonia	3. The sound of the rain falling on the roof has made me feel snug and secure.	Phrases such as “the first winter snowfall” or “a good soap lather when bathing” referred to experiences that do not exist or are not meaningful in this context. Other items, such as “a brisk walk,” “watching sunsets,” “looking at the stars,” “listening to rustling leaves or rain on the roof,” or “petting small animals”, carried cultural connotations that were not necessarily associated with pleasurable experiences. Overall, the broader category of physical anhedonia failed to provide examples that were universally relevant for participants.
WS	Physical anhedonia	5. The beauty of sunsets is greatly overrated.	Phrases such as “the first winter snowfall” or “a good soap lather when bathing” referred to experiences that do not exist or are not meaningful in this context. Other items, such as “a brisk walk,” “watching sunsets,” “looking at the stars,” “listening to rustling leaves or rain on the roof,” or “petting small animals”, carried cultural connotations that were not necessarily associated with pleasurable experiences. Overall, the broader category of physical anhedonia failed to provide examples that were universally relevant for participants.

Instrument	Subscale	Question	Brief Explanations
WS	Physical anhedonia	6. The sound of rustling leaves has never much pleased me.	Phrases such as “the first winter snowfall” or “a good soap lather when bathing” referred to experiences that do not exist or are not meaningful in this context. Other items, such as “a brisk walk,” “watching sunsets,” “looking at the stars,” “listening to rustling leaves or rain on the roof,” or “petting small animals”, carried cultural connotations that were not necessarily associated with pleasurable experiences. Overall, the broader category of physical anhedonia failed to provide examples that were universally relevant for participants.
WS	Physical anhedonia	1. I have often found walks to be relaxing and enjoyable.	Phrases such as “the first winter snowfall” or “a good soap lather when bathing” referred to experiences that do not exist or are not meaningful in this context. Other items, such as “a brisk walk,” “watching sunsets,” “looking at the stars,” “listening to rustling leaves or rain on the roof,” or “petting small animals”, carried cultural connotations that were not necessarily associated with pleasurable experiences. Overall, the broader category of physical anhedonia failed to provide examples that were universally relevant for participants.

Instrument	Subscale	Question	Brief Explanations
WS	Physical anhedonia	10. I don't understand why people enjoy looking at the stars at night.	Phrases such as "the first winter snowfall" or "a good soap lather when bathing" referred to experiences that do not exist or are not meaningful in this context. Other items, such as "a brisk walk," "watching sunsets," "looking at the stars," "listening to rustling leaves or rain on the roof," or "petting small animals", carried cultural connotations that were not necessarily associated with pleasurable experiences. Overall, the broader category of physical anhedonia failed to provide examples that were universally relevant for participants.
WS	Physical anhedonia	11. When I'm feeling a little sad, singing has often made me feel happier	Phrases such as "the first winter snowfall" or "a good soap lather when bathing" referred to experiences that do not exist or are not meaningful in this context. Other items, such as "a brisk walk," "watching sunsets," "looking at the stars," "listening to rustling leaves or rain on the roof," or "petting small animals", carried cultural connotations that were not necessarily associated with pleasurable experiences. Overall, the broader category of physical anhedonia failed to provide examples that were universally relevant for participants.

Instrument	Subscale	Question	Brief Explanations
WS	Physical anhedonia	12. Beautiful scenery has been a great delight to me.	Phrases such as “the first winter snowfall” or “a good soap lather when bathing” referred to experiences that do not exist or are not meaningful in this context. Other items, such as “a brisk walk,” “watching sunsets,” “looking at the stars,” “listening to rustling leaves or rain on the roof,” or “petting small animals”, carried cultural connotations that were not necessarily associated with pleasurable experiences. Overall, the broader category of physical anhedonia failed to provide examples that were universally relevant for participants.
WS	Physical anhedonia	15. Standing on a high place and looking out over the view is very exciting.	Phrases such as “the first winter snowfall” or “a good soap lather when bathing” referred to experiences that do not exist or are not meaningful in this context. Other items, such as “a brisk walk,” “watching sunsets,” “looking at the stars,” “listening to rustling leaves or rain on the roof,” or “petting small animals”, carried cultural connotations that were not necessarily associated with pleasurable experiences. Overall, the broader category of physical anhedonia failed to provide examples that were universally relevant for participants.

Instrument	Subscale	Question	Brief Explanations
WS	Physical anhedonia	4. After a busy day, a slow walk has often felt relaxing.	Phrases such as “the first winter snowfall” or “a good soap lather when bathing” referred to experiences that do not exist or are not meaningful in this context. Other items, such as “a brisk walk,” “watching sunsets,” “looking at the stars,” “listening to rustling leaves or rain on the roof,” or “petting small animals”, carried cultural connotations that were not necessarily associated with pleasurable experiences. Overall, the broader category of physical anhedonia failed to provide examples that were universally relevant for participants.
WS	Physical anhedonia	7. It has often felt good to massage my muscles when they are tired or sore.	Phrases such as “the first winter snowfall” or “a good soap lather when bathing” referred to experiences that do not exist or are not meaningful in this context. Other items, such as “a brisk walk,” “watching sunsets,” “looking at the stars,” “listening to rustling leaves or rain on the roof,” or “petting small animals”, carried cultural connotations that were not necessarily associated with pleasurable experiences. Overall, the broader category of physical anhedonia failed to provide examples that were universally relevant for participants.

Instrument	Subscale	Question	Brief Explanations
WS	Physical anhedonia	8. Flowers aren't as beautiful as many people claim.	Phrases such as "the first winter snowfall" or "a good soap lather when bathing" referred to experiences that do not exist or are not meaningful in this context. Other items, such as "a brisk walk," "watching sunsets," "looking at the stars," "listening to rustling leaves or rain on the roof," or "petting small animals", carried cultural connotations that were not necessarily associated with pleasurable experiences. Overall, the broader category of physical anhedonia failed to provide examples that were universally relevant for participants.
WS	Physical anhedonia	9. I like playing with and petting soft little kittens or puppies.	Phrases such as "the first winter snowfall" or "a good soap lather when bathing" referred to experiences that do not exist or are not meaningful in this context. Other items, such as "a brisk walk," "watching sunsets," "looking at the stars," "listening to rustling leaves or rain on the roof," or "petting small animals", carried cultural connotations that were not necessarily associated with pleasurable experiences. Overall, the broader category of physical anhedonia failed to provide examples that were universally relevant for participants.
WS	Social anhedonia	15. Making new friends isn't worth the energy it takes.	The concept of making friendships as something tiring or costly could not be conveyed meaningfully in this context.

Instrument	Subscale	Question	Brief Explanations
WS	Social anhedonia	2. I never had really close friends in high school.	The concept of high school was replaced with adolescence to be more meaningful in this context. However, participants did not understand the distinction between having close relationships in the past versus the present. We therefore simplified the item to ask directly whether the person enjoys being with their friends or close relationships.
WS	Social anhedonia	8. People who try to get to know me better usually give up after a while.	The item “People who try to get to know me better usually give up after a while” was difficult to adapt. Participants tended to interpret it concretely, in terms of others being too busy or unavailable at a given moment, rather than as a reflection of a permanent social trait or behaviors.
WS	Social anhedonia	6. I prefer hobbies and leisure activities that do not involve other people.	We found it difficult to convey the concept of “hobbies” or “leisure activities”, particularly those that would involve spending time alone. The idea of “time off” was not very meaningful in this context. Instead, leisure activities were generally understood as social activities shared with others, such as playing football or attending chicha parties.

Table SM9. Complete list of "original" items abandoned for low reliability reasons.

Abbreviations: ASRS = Adult ADHD Self-Report; AQ = Autism Quotient; B2 = Cross-Cultural Big Two; BAPQ = Broad Autism Phenotype Questionnaire; BFI = Big Five Inventory; CATI = Comprehensive Autistic Trait Inventory; DS-Tsi = Depressive Symptoms (Tsimane'); GAD = Generalized Anxiety Disorder; IMHA = International Mental Health Assessment; OCI-R = Obsessive-Compulsive Inventory–Revised; SPQ = Schizotypal Personality Questionnaire; WS = Wisconsin Scales of Vulnerability to Psychosis–Brief.

Instrument	Subscale	Question	Brief Explanations
ASRS	Hyperactivity-Impulsivity	18. How often do you interrupt others when they are busy?	Some problems with reverse coding
ASRS	Inattention	9. How often do you have difficulty concentrating on what people say to you, even when they are speaking to you directly?	Some problems with reverse coding
AQ	Imagination	14 I find making up stories easy	Low Kappa & comprehension problems
BFI	Agreeableness	I see myself as someone who tends to be judgmental	Interviewers concerned about honesty
BAPQ	Pragmatic Language	29. I leave long pauses in conversation.	Low variation
CATI	Sensory sensitivity	18. I am oversensitive to touch	Low variation & reverse coding problems
B2	Social Self-Regulation	20. I tend to be bad	Low Kappa. Interviewers concerned about honesty
B2	Social Self-Regulation	21. I tend to be disobedient	Low variation
B2	Social Self-Regulation	18.I tend to be aggressive	Interviewers concerned about honesty
B2	Social Self-Regulation	22. I tend to be gossipy	Interviewers concerned about honesty
B2	Social Self-Regulation	5. I tend to be faithful	Interviewers concerned about honesty

Instrument	Subscale	Question	Brief Explanations
DS-Tsi	-	[item in Tsimane - Do you criticize yourself for your flaws?"]	Low variation & reverse coding problems
DS-Tsi	-	[item in Tsimane - “Do you feel that you lose interest in sex?"]	Interviewers concerned about honesty
IMHA	Anger Issues	5.I got so angry that I threatened someone.	Low Kappa & reverse coding problems
IMHA	Anger Issues	6.I got so angry that I broke something.	Low variation
IMHA	Anxiety	8.I had difficulty breathing (not due to a medical condition).	Some problems with reverse coding and comprehension
IMHA	Substance Abuse	5.Family or friends suggested I should cut down on my drinking or drug use.	Low variation & reverse coding problems
IMHA	Sleep issues	s3.Not getting enough sleep interferes with my daily activities.	Low Kappa & comprehension problems
IMHA	Anger Issues	4.I expressed anger at someone I encountered in public/a stranger.	Interviewers concerned about honesty
IMHA	Substance Abuse	2.I drank enough to pass out.	Interviewers concerned about honesty
IMHA	Substance Abuse	4.I felt guilt or remorse after drinking or using drugs.	Interviewers concerned about honesty
SPQ	Constricted affect	26. I rarely laugh and smile.	Low variation & reverse coding problems
SPQ	Excessive social anxiety	46. I feel very uncomfortable in social situations involving unfamiliar people.	Low Kappa & comprehension problems

Instrument	Subscale	Question	Brief Explanations
SPQ	No Closed Friends	62. I attach little importance to having close friends.	Low variation
SPQ	Odd Beliefs or Magical Thinking	30. Do you believe in clairvoyancy (psychic forces, fortune telling)?	Low Kappa & comprehension problems
SPQ	Odd Speech	25. I sometimes forget what I am trying to say.	Low Kappa & comprehension problems
SPQ	Unusual Perceptual Experiences	13. Have you ever had the sense that some person or force is around you, even though you cannot see anyone?	Low Kappa & comprehension problems
SPQ	Unusual Perceptual Experiences	61. Do you ever suddenly feel distracted by distant sounds that you are not normally aware of?	Low Kappa & comprehension problems
WS	Social anhedonia	14. When things are going really well for my close friends, it makes me feel good too.	Low Kappa & comprehension problems

Table SM10. “Original” items removed because of redundancy with retained questions

Abbreviations: ASRS = Adult ADHD Self-Report; AQ = Autism Quotient; B2 = Cross-Cultural Big Two; BAPQ = Broad Autism Phenotype Questionnaire; BFI = Big Five Inventory; CATI = Comprehensive Autistic Trait Inventory; DS-Tsi = Depressive Symptoms (Tsimane’); GAD = Generalized Anxiety Disorder; IMHA = International Mental Health Assessment; OCI-R = Obsessive-Compulsive Inventory–Revised; SPQ = Schizotypal Personality Questionnaire; WS = Wisconsin Scales of Vulnerability to Psychosis–Brief.

Redundancy was evaluated with consideration of rephrasings, scripted prompts, and potential examples. Accordingly, the “original” English wording may not fully reflect the item ultimately judged to be redundant.

Instrument	Subscale	Question	# in final instrument	Instrument_1	Subscale_1	# in original scale_1	Question_1	# in final instrument 2	Instrument_2	Subscale_2	# in original scale_2	Question_2
AQ	Social skill	44 I enjoy social occasions	9	AQ	Social skill	11	I find social situations easy	NA	NA	NA	NA	NA
AQ	Attention to detail	28 I usually concentrate more on the whole picture, rather than the small details	10	AQ	Attention to detail	12	I tend to notice details that others do not	NA	NA	NA	NA	NA
AQ	Attention to detail	30 I don't usually notice small changes in a situation, or a person's appearance	10	AQ	Attention to detail	12	I tend to notice details that others do not	NA	NA	NA	NA	NA
AQ	Attention to detail	6 I usually notice car number plates or similar strings of information	10	AQ	Attention to detail	12	I tend to notice details that others do not	NA	NA	NA	NA	NA
AQ	Communication	38 I am good at social chit-chat	12	AQ	Communication	17	I enjoy social chit-chat	NA	NA	NA	NA	NA
AQ	Imagination	50 I find it easy to play games with children that involve pretending	19	AQ	Imagination	40	When younger, I enjoyed playing games involving pretending with other children	NA	NA	NA	NA	NA
AQ	Attention switching	4 I frequently get strongly absorbed in one thing	58	BFI	Conscientiousness	42	I see myself as someone who is easily distracted	NA	NA	NA	NA	NA
AQ	Communication	39 People tell me that I keep going on and on about the same thing	63	BAPQ	Pragmatic Language	17	I have been told that I talk too much about certain topics.	NA	NA	NA	NA	NA
AQ	Social skill	1 I prefer to do things with others rather than on my own	66	BAPQ	Aloof	31	I prefer to be alone rather than with others.	NA	NA	NA	NA	NA

AQ	Social skill	13 I would rather go to a library than a party	66	BAPQ	Aloof	31	I prefer to be alone rather than with others.	NA	NA	NA	NA	NA
AQ	Social skill	15 I find myself drawn more strongly to people than to things	66	BAPQ	Aloof	31	I prefer to be alone rather than with others.	NA	NA	NA	NA	NA
AQ	Social skill	36 I find it easy to work out what someone is thinking or feeling just by looking at their face	70	CATI	Communication	19	I can tell how people feel from their facial expressions	NA	NA	NA	NA	NA
AQ	Attention switching	2 I prefer to do things the same way over and over again	76	CATI	Cognitive rigidity	5	There are certain activities that I always choose to do the same way, every time	NA	NA	NA	NA	NA
AQ	Attention switching	25 It does not upset me if my daily routine is disturbed	71	CATI	Cognitive rigidity	21	I feel discomfort when prevented from completing a particular routine	NA	NA	NA	NA	NA
AQ	Social skill	47 I enjoy meeting new people	103	SPQ	Excessive social anxiety	29	I get anxious when meeting people for the first time.	NA	NA	NA	NA	NA
ASRS	Hyperactivity-Impulsivity	6. How often do you feel overly active and compelled to do things, like you were driven by a motor?	36	B2	Dynamism	37	I tend to be lively	NA	NA	NA	NA	NA
ASRS	Hyperactivity-Impulsivity	15. How often do you find yourself talking too much when you are in social situations	45	BFI	Extraversion	16	I see myself as someone who tends to be quiet	NA	NA	NA	NA	NA
ASRS	Hyperactivity-	13. How often	56	BFI	Neuroticism	38	I see myself as	NA	NA	NA	NA	NA

	Impulsivity	do you feel restless or fidgety?					someone who gets nervous easily					
ASRS	Inattention	8. How often do you have difficulty keeping your attention when you are doing boring or repetitive work?	58	BFI	Conscientiousness	42	I see myself as someone who is easily distracted	NA	NA	NA	NA	NA
ASRS	Inattention	11. How often are you distracted by activity or noise around you?	58	BFI	Conscientiousness	42	I see myself as someone who is easily distracted	NA	NA	NA	NA	NA
B2	Social Self-Regulation	16.I tend to be polite	28	B2	Social Self-Regulation	17	I tend to be respectful	NA	NA	NA	NA	NA
B2	Social Self-Regulation	3. I tend to be courteous	28	B2	Social Self-Regulation	17	I tend to be respectful	NA	NA	NA	NA	NA
B2	Social Self-Regulation	12. I tend to be modest	29	B2	Social Self-Regulation	19	I tend to be arrogant	NA	NA	NA	NA	NA
B2	Dynamism	38. I tend to be cowardly	32	B2	Dynamism	26	I tend to be bold	NA	NA	NA	NA	NA
B2	Dynamism	27. I tend to be brave	32	B2	Dynamism	26	I tend to be bold	NA	NA	NA	NA	NA
B2	Dynamism	31.I tend to be courageous	32	B2	Dynamism	26	I tend to be bold	NA	NA	NA	NA	NA
B2	Dynamism	32. I tend to be daring	32	B2	Dynamism	26	I tend to be bold	NA	NA	NA	NA	NA
B2	Dynamism	46. I tend to be unhappy	35	B2	Dynamism	36	I tend to be happy	38	B2	Dynamism	42	I tend to be sad
B2	Dynamism	25.I tend to be active	36	B2	Dynamism	37	I tend to be lively	NA	NA	NA	NA	NA
B2	Dynamism	47. I tend to be weak	36	B2	Dynamism	37	I tend to be lively	NA	NA	NA	NA	NA
B2	Dynamism	34. I tend to be dynamic	36	B2	Dynamism	37	I tend to be lively	NA	NA	NA	NA	NA
B2	Dynamism	35.I tend to be energetic	36	B2	Dynamism	37	I tend to be lively	NA	NA	NA	NA	NA
B2	Dynamism	28. I tend to be cheerful	35	B2	Dynamism	36	I tend to be happy	38	B2	Dynamism	42	I tend to be sad
B2	Dynamism	45. I tend to be	39	B2	Dynamism	43	I tend to be shy	NA	NA	NA	NA	NA

		timid										
B2	Social Self-Regulation	10. I tend to be hardworking	44	BFI	Conscientiousness	14	I see myself as someone who is a reliable worker, trustworthy.	NA	NA	NA	NA	NA
B2	Dynamism	41. I tend to be quiet	45	BFI	Extraversion	16	I see myself as someone who tends to be quiet	NA	NA	NA	NA	NA
B2	Dynamism	44.I tend to be silent	45	BFI	Extraversion	16	I see myself as someone who tends to be quiet	NA	NA	NA	NA	NA
B2	Social Self-Regulation	13. I tend to be naive	48	BFI	Sociability	24	I see myself as someone who is generally trusting	NA	NA	NA	NA	NA
B2	Social Self-Regulation	4. I tend to be diligent	52	BFI	Conscientiousness	3	I see myself as someone who is meticulous at work	44	BFI	Conscientiousness	14	I see myself as someone who is a reliable worker, trustworthy.
B2	Social Self-Regulation	8. I tend to be gentle	55	BFI	Agreeableness	37	I see myself as someone who is considerate and kind to almost everyone	NA	NA	NA	NA	NA
B2	Social Self-Regulation	9.I tend to be good-natured	55	BFI	Agreeableness	37	I see myself as someone who is considerate and kind to almost everyone	NA	NA	NA	NA	NA
B2	Dynamism	39. I tend to be a loner	66	BAPQ	Aloof	31	I prefer to be alone rather than with others.	NA	NA	NA	NA	NA
B2	Dynamism	33.I tend to be determined	57	BFI	Extraversion	40	I see myself as someone who is assertive, not afraid to express what I want	51	BFI	Conscientiousness	29	I see myself as someone who does things efficiently

B2	Social Self-Regulation	1. I tend to be calm	43	BFI	Sociability	13	I see myself as someone who starts disputes with others	NA	NA	NA	NA	NA
B2	Social Self-Regulation	15. I tend to be peaceful	43	BFI	Sociability	13	I see myself as someone who starts disputes with others	NA	NA	NA	NA	NA
B2	Social Self-Regulation	14. I tend to be obedient	44	BFI	Conscientiousness	14	I see myself as someone who is a reliable worker, trustworthy.	NA	NA	NA	NA	NA
BAPQ	Aloof	9. I enjoy being in social situations.	9	AQ	Social skill	11	I find social situations easy	NA	NA	NA	NA	NA
BAPQ	Aloof	27. Conversation bores me.	12	AQ	Communication	17	I enjoy social chit-chat	NA	NA	NA	NA	NA
BAPQ	Aloof	36. I enjoy chatting with people.	12	AQ	Communication	17	I enjoy social chit-chat	NA	NA	NA	NA	NA
BAPQ	Pragmatic Language	21. I can tell when someone is not interested in what I am saying.	14	AQ	Communication	31	I know how to tell if someone listening to me is getting bored	NA	NA	NA	NA	NA
BAPQ	Aloof	12. People find it easy to approach me.	54	BFI	Agreeableness	33	I see myself as someone who is sometimes cold and distant	NA	NA	NA	NA	NA
BAPQ	Aloof	28. I am warm and friendly in my interactions with others.	55	BFI	Agreeableness	37	I see myself as someone who is considerate and kind to almost everyone	NA	NA	NA	NA	NA
BAPQ	Pragmatic Language	2. I find it hard to get my words out smoothly.	62	BAPQ	Pragmatic Language	14	People ask me to repeat things I’ve said because they don’t understand.	NA	NA	NA	NA	NA
BAPQ	Rigid	6. People have to talk me into	64	BAPQ	Rigid	19	I look forward to trying new	NA	NA	NA	NA	NA

		trying something new.					things.					
BAPQ	Aloof	1. I like being around other people.	66	BAPQ	Aloof	31	I prefer to be alone rather than with others.	NA	NA	NA	NA	NA
BAPQ	Rigid	13. I feel a strong need for sameness from day to day.	71	CATI	Cognitive rigidity	21	I feel discomfort when prevented from completing a particular routine	NA	NA	NA	NA	NA
BAPQ	Rigid	22. I have a hard time dealing with changes in my routine.	71	CATI	Cognitive rigidity	21	I feel discomfort when prevented from completing a particular routine	NA	NA	NA	NA	NA
BAPQ	Rigid	30. I alter my daily routine by trying something different.	71	CATI	Cognitive rigidity	21	I feel discomfort when prevented from completing a particular routine	NA	NA	NA	NA	NA
BAPQ	Rigid	24. I act very set in my ways.	76	CATI	Cognitive rigidity	5	There are certain activities that I always choose to do the same way, every time	NA	NA	NA	NA	NA
BAPQ	Rigid	35. I keep doing things the way I know, even if another way might be better.	76	CATI	Cognitive rigidity	5	There are certain activities that I always choose to do the same way, every time	NA	NA	NA	NA	NA
BAPQ	Rigid	15. I am flexible about how things should be done.	76	CATI	Cognitive rigidity	5	There are certain activities that I always choose to do the same way, every time	NA	NA	NA	NA	NA
BAPQ	Rigid	33. I like to closely follow a	76	CATI	Cognitive rigidity	5	There are certain	NA	NA	NA	NA	NA

		routine while working.					activities that I always choose to do the same way, every time					
BAPQ	Aloof	16. I look forward to situations where I can meet new people.	103	SPQ	Excessive social anxiety	29	I get anxious when meeting people for the first time.	NA	NA	NA	NA	NA
BAPQ	Pragmatic Language	32. I lose track of my original point when talking to people.	105	SPQ	Odd Speech	34	I often ramble on too much when speaking.	NA	NA	NA	NA	NA
BFI	Conscientiousness	21. I see myself as someone who perseveres until the job is done	1	ASRS	Inattention	1	How often do you have trouble wrapping up the fine details of a project, once the challenging parts have been done?	NA	NA	NA	NA	NA
BFI	Conscientiousness	34. I see myself as someone who makes plans and follows them carefully	16	AQ	Attention switching	34	I enjoy doing things spontaneously	NA	NA	NA	NA	NA
BFI	Agreeableness	22. I see myself as someone who is sometimes rude to others	28	B2	Social Self-Regulation	17	I tend to be respectful	NA	NA	NA	NA	NA
BFI	Openness	23. I see myself as someone who is inventive	33	B2	Dynamism	29	I tend to be clever	NA	NA	NA	NA	NA
BFI	Openness	36. I see myself as someone who likes to reflect, play with ideas	33	B2	Dynamism	29	I tend to be clever	NA	NA	NA	NA	NA
BFI	Openness	20. I see myself as someone who has an	33	B2	Dynamism	29	I tend to be clever	NA	NA	NA	NA	NA

		active imagination										
BFI	Openness	31. I see myself as someone who is witty, analytical	33	B2	Dynamism	29	I tend to be clever	NA	NA	NA	NA	NA
BFI	Openness	5. I see myself as someone who is original, comes up with new ideas	33	B2	Dynamism	29	I tend to be clever	NA	NA	NA	NA	NA
BFI	Extraversion	11. I see myself as someone who is full of energy	36	B2	Dynamism	37	I tend to be lively	NA	NA	NA	NA	NA
BFI	Extraversion	32. I see myself as someone who radiates enthusiasm	36	B2	Dynamism	37	I tend to be lively	NA	NA	NA	NA	NA
BFI	Neuroticism	4. I see myself as someone who is depressive, melancholic	35	B2	Dynamism	36	I tend to be happy	38	B2	Dynamism	42	I tend to be sad
BFI	Extraversion	27. I see myself as someone who is sometimes shy, inhibited	39	B2	Dynamism	43	I tend to be shy	NA	NA	NA	NA	NA
BFI	Agreeableness	7. I see myself as someone who is generous and helps others	41	B2	Social Self-Regulation	7	I tend to be generous	NA	NA	NA	NA	NA
BFI	Conscientiousness	25. I see myself as someone who tends to be lazy	44	BFI	Conscientiousness	14	I see myself as someone who is a reliable worker, trustworthy.	NA	NA	NA	NA	NA
BFI	Extraversion	1. I see myself as someone who is very talkative	45	BFI	Extraversion	16	I see myself as someone who tends to be quiet	NA	NA	NA	NA	NA
BFI	Openness	44. I see myself as someone who has few	46	BFI	Openness	17	I see myself as someone who values the	NA	NA	NA	NA	NA

		artistic interests					artistic, the aesthetic					
BFI	Conscientiousness	8. I see myself as someone who can sometimes be somewhat careless	52	BFI	Conscientiousness	3	I see myself as someone who is meticulous at work	NA	NA	NA	NA	NA
BFI	Neuroticism	19. I see myself as someone who is emotionally stable, difficult to upset	53	BFI	Neuroticism	30	I see myself as someone who is temperamental, moody	31	B2	Social Self-Regulation	24	I tend to be hot-tempered
BFI	Neuroticism	15. I see myself as someone who often gets tense	56	BFI	Neuroticism	38	I see myself as someone who gets nervous easily	NA	NA	NA	NA	NA
BFI	Neuroticism	35. I see myself as someone who stays calm in difficult situations.	49	BFI	Neuroticism	26	I see myself as someone who cares a lot about things	78	DS-Tsi	--	11	In the past month, have you had problems sleeping?
BFI	Neuroticism	9. I see myself as someone who is calm, manages stress well	43	BFI	Sociability	13	I see myself as someone who starts disputes with others	49	BFI	Neuroticism	26	I see myself as someone who cares a lot about things
CATI	Social interactions	15. Social interaction is easy for me	9	AQ	Social skill	11	I find social situations easy	NA	NA	NA	NA	NA
CATI	Social interactions	17. I find social interactions stressful	9	AQ	Social skill	11	I find social situations easy	NA	NA	NA	NA	NA
CATI	Social interactions	30. Social occasions are often challenging for me	9	AQ	Social skill	11	I find social situations easy	NA	NA	NA	NA	NA
CATI	Social interactions	8. I generally enjoy social events	9	AQ	Social skill	11	I find social situations easy	NA	NA	NA	NA	NA
CATI	Cognitive rigidity	34. It annoys me when plans I have made	65	BAPQ	Rigid	3	I am comfortable with	NA	NA	NA	NA	NA

		are changed					unexpected changes in plans.					
CATI	Cognitive rigidity	38. I like to arrange items in rows or patterns	69	CATI	Cognitive rigidity	14	I like my belongings to be sorted in certain ways and will spend time making sure they are that way	NA	NA	NA	NA	NA
CATI	Communication	23. I find it easy to sense what someone else is feeling	70	CATI	Communication	19	I can tell how people feel from their facial expressions	NA	NA	NA	NA	NA
CATI	Communication	13. Reading non-verbal cues (e.g., facial expressions, body language) is difficult for me	70	CATI	Communication	19	I can tell how people feel from their facial expressions	NA	NA	NA	NA	NA
CATI	Communication	26. I rarely use non-verbal cues in my interactions with others	70	CATI	Communication	19	I can tell how people feel from their facial expressions	NA	NA	NA	NA	NA
CATI	Cognitive rigidity	2. I like to stick to certain routines for everyday tasks	71	CATI	Cognitive rigidity	21	I feel discomfort when prevented from completing a particular routine	NA	NA	NA	NA	NA
CATI	Repetitive behaviour	41. I have certain habits that I find difficult to stop (e.g., biting/tearing nails, pulling strands of hair)	74	CATI	Repetitive behaviour	32	There are certain repetitive actions that others consider to be ’characteristic’ of me (e.g. stroking my hair)	NA	NA	NA	NA	NA
CATI	Repetitive behaviour	1. I often find myself fiddling or playing	74	CATI	Repetitive behaviour	32	There are certain repetitive	NA	NA	NA	NA	NA

		repetitively with objects (e.g., clicking pens)					actions that others consider to be 'characteristic' of me (e.g. stroking my hair)					
CATI	Repetitive behaviour	20. I have a tendency to pace or move around in a repetitive path	74	CATI	Repetitive behaviour	32	There are certain repetitive actions that others consider to be 'characteristic' of me (e.g. stroking my hair)	NA	NA	NA	NA	NA
CATI	Repetitive behaviour	7. I often rock when sitting in a chair	74	CATI	Repetitive behaviour	32	There are certain repetitive actions that others consider to be 'characteristic' of me (e.g. stroking my hair)	NA	NA	NA	NA	NA
CATI	Communication	42. I have difficulty understanding the 'unspoken rules' of social situations	77	CATI	Social camouflage	6	Sometimes I watch people interacting and try to copy them when I need to socialise	NA	NA	NA	NA	NA
CATI	Social camouflage	22. I rely on a set of scripts when I talk with people	77	CATI	Social camouflage	6	Sometimes I watch people interacting and try to copy them when I need to socialise	NA	NA	NA	NA	NA
CATI	Social camouflage	29. Before engaging in a social situation, I will create a script to follow where possible	77	CATI	Social camouflage	6	Sometimes I watch people interacting and try to copy them when I need to socialise	NA	NA	NA	NA	NA

CATI	Social camouflage	39. I try to follow certain ’rules’ in order to get by in social situations	77	CATI	Social camouflage	6	Sometimes I watch people interacting and try to copy them when I need to socialise	NA	NA	NA	NA	NA
CATI	Social interactions	10. In social situations, I try to avoid interactions with other people	103	SPQ	Excessive social anxiety	29	I get anxious when meeting people for the first time.	NA	NA	NA	NA	NA
CATI	Social interactions	28. I am confident and capable when meeting new people	103	SPQ	Excessive social anxiety	29	I get anxious when meeting people for the first time.	NA	NA	NA	NA	NA
DS-Tsi	DP7	[item in Tsimane - Roughly translates to "Do you feel tired or weak?"]	36	B2	Dynamism	37	I tend to be lively	NA	NA	NA	NA	NA
DS-Tsi	DP6	[item in Tsimane - Roughly translates to “Do you feel you have no interest in anything ?"]	36	B2	Dynamism	37	I tend to be lively	NA	NA	NA	NA	NA
DS-Tsi	DP14	[item in Tsimane - Roughly translates to “Do you lose confidence in yourself or hope for your future?"]	37	B2	Dynamism	40	I tend to be pessimistic	34	B2	Dynamism	30	I tend to be confident
DS-Tsi	DP1	[item in Tsimane - Roughly translates to "Do you feel sad or helpless?"]	35	B2	Dynamism	36	I tend to be happy	38	B2	Dynamism	42	I tend to be sad

DS-Tsi	DP10	[item in Tsimane - Roughly translates to "Do you distrust others? "]	48	BFI	Sociability	24	I see myself as someone who is generally trusting	NA	NA	NA	NA	NA
DS-Tsi	DP17	[item in Tsimane - Roughly translates to "Do you get angry easily or feel moody at times?"]	53	BFI	Neuroticism	30	I see myself as someone who is temperamental, moody	31	B2	Social Self-Regulation	24	I tend to be hot-tempered
DS-Tsi	DP9	[item in Tsimane - Roughly translates to "Do you feel nervous? Do you worry about everything and feel afraid?"]	56	BFI	Neuroticism	38	I see myself as someone who gets nervous easily	49	BFI	Neuroticism	26	I see myself as someone who cares a lot about things
DS-Tsi	DP15	[item in Tsimane - Roughly translates to "Do you feel that you have difficulties making decisions?"]	57	BFI	Extraversion	40	I see myself as someone who is assertive, not afraid to express what I want	NA	NA	NA	NA	NA
DS-Tsi	DP8	[item in Tsimane - Roughly translates to "Do you find it hard to concentrate?"]	58	BFI	Conscientiousness	42	I see myself as someone who is easily distracted	NA	NA	NA	NA	NA
DS-Tsi	PR3	[item in Tsimane - Roughly translates to "Is it difficult to sleep because you are worried?"]	78	DS-Tsi	--	11	[item in Tsimane - Roughly translates to “Do you have problems with sleep or see changes in your sleep?”]	NA	NA	NA	NA	NA

DS-Tsi	DP16	[item in Tsimane - Roughly translates to "Do you feel as though you deserve punishment, or that you are lucky but do not deserve it?"]	82	DS-Tsi	--	5	[item in Tsimane - Roughly translates to “Do you feel like your are a worthless person”]	NA	NA	NA	NA	NA
DS-Tsi	DP18	[item in Tsimane - Roughly translates to "Do you get ideas stuck in your head and cannot free yourself from them?"]	96	OCI-R	--	18	How much has frequently getting nasty thoughts and having difficulty in getting rid of them distressed or bothered you during the past month?	NA	NA	NA	NA	NA
GAD	NA	5. Being so restless that it is hard to sit still	3	ASRS	Hyperactivity-Impulsivity	12	How often do you leave your seat in meetings or other situations in which you are expected to remain seated?	NA	NA	NA	NA	NA
GAD	NA	1. Feeling nervous, anxious, or on edge	49	BFI	Neuroticism	26	I see myself as someone who cares a lot about things	56	BFI	Neuroticism	38	I see myself as someone who gets nervous easily
GAD	NA	3. Worrying too much about different things	49	BFI	Neuroticism	26	I see myself as someone who cares a lot about things	56	BFI	Neuroticism	38	I see myself as someone who gets nervous easily
GAD	NA	6. Becoming easily annoyed or irritable	53	BFI	Neuroticism	30	I see myself as someone who is temperamental, moody	31	B2	Social Self-Regulation	24	I tend to be hot-tempered
GAD	NA	7. Feeling afraid, as if	84	IMHA	Anxiety	1	I felt afraid.	NA	NA	NA	NA	NA

		something awful might happen										
GAD	NA	2. Not being able to stop or control worrying	96	OCI-R	--	18	How much has frequently getting nasty thoughts and having difficulty in getting rid of them distressed or bothered you during the past month?	NA	NA	NA	NA	NA
IMHA	ADHD	2. I had problems remembering appointments or obligations.	6	ASRS	Inattention	3	How often do you have problems remembering appointments or obligations?	NA	NA	NA	NA	NA
IMHA	ADHD	3. I fidgeted or squirmed with my hands or my feet when I had to sit down for a long time.	7	ASRS	Hyperactivity-Impulsivity	5	How often do you fidget or squirm with your hands or feet when you have to sit down for a long time?	NA	NA	NA	NA	NA
IMHA	Depression	6. I lacked confidence in myself.	34	B2	Dynamism	30	I tend to be confident	NA	NA	NA	NA	NA
IMHA	Depression	4. I felt sad and unhappy.	35	B2	Dynamism	36	I tend to be happy	38	B2	Dynamism	42	I tend to be sad
IMHA	ADHD	4. I felt overly active and compelled to do things, like I was driven by a motor.	36	B2	Dynamism	37	I tend to be lively	NA	NA	NA	NA	NA
IMHA	Depression	2. I lost interest or pleasure in doing things I used to like.	36	B2	Dynamism	37	I tend to be lively	NA	NA	NA	NA	NA
IMHA	Depression	5. I felt hopeless about the future.	37	B2	Dynamism	40	I tend to be pessimistic	NA	NA	NA	NA	NA
IMHA	Depression	3. I felt distant	54	BFI	Agreeableness	33	I see myself as	NA	NA	NA	NA	NA

		or cut off from other people.					someone who is sometimes cold and distant					
IMHA	PTSD	2. I felt jumpy or easily startled.	56	BFI	Neuroticism	38	I see myself as someone who gets nervous easily	NA	NA	NA	NA	NA
IMHA	Depression	1. I had difficulty making decisions.	57	BFI	Extraversion	40	I see myself as someone who is assertive, not afraid to express what I want	NA	NA	NA	NA	NA
IMHA	Sleep issues	1. I had difficulty falling asleep.	78	DS-Tsi	--	11	[item in Tsimane - Roughly translates to “Do you have problems with sleep or see changes in your sleep?”]	NA	NA	NA	NA	NA
IMHA	Sleep issues	2. I woke up too early, or had trouble staying asleep.	78	DS-Tsi	--	11	[item in Tsimane - Roughly translates to “Do you have problems with sleep or see changes in your sleep?”]	NA	NA	NA	NA	NA
IMHA	Depression	8. I thought I would be better off dead or thought about killing myself.	81	DS-Tsi	--	4	[item in Tsimane - Roughly translates to “Do you think of hurting or killing yourself?”]	NA	NA	NA	NA	NA
IMHA	Depression	7. I felt guilty or had a bad conscience.	82	DS-Tsi	--	5	[item in Tsimane - Roughly translates to “Do you feel like your are a worthless person”]	NA	NA	NA	NA	NA

IMHA	Sleep issues	4. When I got up in the morning, I did not feel well-rested.	36	B2	Dynamism	37	I tend to be lively	NA	NA	NA	NA	NA
IMHA	Anger Issues	1. I felt angry with someone at work, at school, or at church.	83	IMHA	Anger Issues	2	I argued with someone at work, at school, or at church.	NA	NA	NA	NA	NA
IMHA	Anger Issues	3. I yelled at someone at work, at school, or at church.	83	IMHA	Anger Issues	2	I argued with someone at work, at school, or at church.	NA	NA	NA	NA	NA
IMHA	Life stress	3. Someone I'm close to made me feel badly about myself.	83	IMHA	Anger Issues	2	I argued with someone at work, at school, or at church.	NA	NA	NA	NA	NA
IMHA	Life stress	1. I argued with friends or family.	83	IMHA	Anger Issues	2	I argued with someone at work, at school, or at church.	NA	NA	NA	NA	NA
IMHA	Life stress	2. A friend, partner, or family member treated me poorly.	83	IMHA	Anger Issues	2	I argued with someone at work, at school, or at church.	NA	NA	NA	NA	NA
IMHA	Relationship	2. My partner insulted me or made me feel bad about myself.	83	IMHA	Anger Issues	2	I argued with someone at work, at school, or at church.	NA	NA	NA	NA	NA
IMHA	Life stress	3. I got bad news about something important in my life.	87	IMHA	Life stress	1	Stressful things (family illness, financial set backs, accidents) occurred in my life.	NA	NA	NA	NA	NA
IMHA	Life stress	5. Medical issues (my own or those of a close other) caused stress in my life.	87	IMHA	Life stress	1	Stressful things (family illness, financial set backs, accidents) occurred in my life.	NA	NA	NA	NA	NA

IMHA	Relationship	4. My romantic partner threatened me.	89	IMHA	Relationship	3	I felt afraid of my romantic partner.	NA	NA	NA	NA	NA
IMHA	Substance Abuse	7. I drank alcohol to the degree that I felt intoxicated	91	IMHA	Substance Abuse	1	I thought I should cut down on my drinking or drug use.	NA	NA	NA	NA	NA
IMHA	Substance Abuse	3. I forgot things I did while using alcohol or drugs.	92	IMHA	Substance Abuse	6	I failed to do what was normally expected from me because of drinking or drug use.	NA	NA	NA	NA	NA
IMHA	Anxiety	3. It was hard to control my worrying.	96	OCI-R	--	18	How much has frequently getting nasty thoughts and having difficulty in getting rid of them distressed or bothered you during the past month?	NA	NA	NA	NA	NA
IMHA	Psychosis	1. I heard things other people can't hear, such as voices, even when no one was around.	108	SPQ	Unusual Perceptual Experiences	4	Have you often mistaken objects or shadows for people, or noises for voices?	NA	NA	NA	NA	NA
IMHA	Psychosis	2. I felt that someone could hear my thoughts, or that I could hear what another person was thinking.	111	SPQ	Odd Beliefs or Magical Thinking	55	Have you ever felt that you are communicating with another person telepathically (by mind-reading)?	NA	NA	NA	NA	NA
OCI-R	NA	17. How much has washing your hands more often and longer than necessary distressed or	93	OCI-R	--	11	How much has sometimes having to wash or clean yourself simply because you feel	NA	NA	NA	NA	NA

		bothered you during the past month?					contaminated distressed or bothered you during the past month?					
OCI-R	NA	9. How much has getting upset if others change the way you have arranged things distressed or bothered you during the past month?	95	OCI-R	--	15	How much do you need things to be arranged in a particular order, and how much has this distressed or bothered you during the past month?	NA	NA	NA	NA	NA
OCI-R	NA	3. How much has getting upset if objects are not arranged properly distressed or bothered you during the past month?	95	OCI-R	--	15	How much do you need things to be arranged in a particular order, and how much has this distressed or bothered you during the past month?	NA	NA	NA	NA	NA
OCI-R	NA	6. How much has finding it difficult to control your own thoughts distressed or bothered you during the past month?	96	OCI-R	--	18	How much has frequently getting nasty thoughts and having difficulty in getting rid of them distressed or bothered you during the past month?	NA	NA	NA	NA	NA
OCI-R	NA	12. How much has being upset by unpleasant thoughts that come into your mind against your will distressed or bothered you during the past month?	96	OCI-R	--	18	How much has frequently getting nasty thoughts and having difficulty in getting rid of them distressed or bothered you during the past month?	NA	NA	NA	NA	NA

OCI-R	NA	1. How much has saving up so many things that they get in the way, distressed or bothered you during the past month?	98	OCI-R	--	7	How much has collecting things you don't need distressed or bothered you during the past month?	NA	NA	NA	NA	NA
SPQ	No Closed Friends	57. I tend to keep in the background on social occasions.	39	B2	Dynamism	43	I tend to be shy	NA	NA	NA	NA	NA
SPQ	No Closed Friends	24. I am mostly quiet when with other people.	45	BFI	Extraversion	16	I see myself as someone who tends to be quiet	NA	NA	NA	NA	NA
SPQ	Suspiciousness	27. Do you sometimes get concerned that friends or coworkers are not really loyal or trustworthy?	48	BFI	Sociability	24	I see myself as someone who is generally trusting	NA	NA	NA	NA	NA
SPQ	Suspiciousness	36. I feel I have to be on my guard even with friends.	48	BFI	Sociability	24	I see myself as someone who is generally trusting	NA	NA	NA	NA	NA
SPQ	Constricted affect	8. People sometimes find me aloof and distant.	54	BFI	Agreeableness	33	I see myself as someone who is sometimes cold and distant	NA	NA	NA	NA	NA
SPQ	No Closed Friends	66. Do you feel that you cannot get "close" to people?	54	BFI	Agreeableness	33	I see myself as someone who is sometimes cold and distant	NA	NA	NA	NA	NA
SPQ	Constricted affect	73. I tend to keep my feelings to myself.	60	BFI	Extraversion	6	I see myself as someone who is reserved	NA	NA	NA	NA	NA
SPQ	No Closed Friends	15. I prefer to keep myself to myself.	60	BFI	Extraversion	6	I see myself as someone who is reserved	NA	NA	NA	NA	NA
SPQ	No Closed Friends	33. I find it hard	60	BFI	Extraversion	6	I see myself as	NA	NA	NA	NA	NA

		to be emotionally close to other people.					someone who is reserved					
SPQ	Suspiciousness	52. Have you found that it is best not to let other people know too much about you?	60	BFI	Extraversion	6	I see myself as someone who is reserved	NA	NA	NA	NA	NA
SPQ	Constricted affect	68. I do not have an expressive and lively way of speaking.	61	BAPQ	Pragmatic Language	10	My voice has a flat or monotone sound to it.	NA	NA	NA	NA	NA
SPQ	Odd Speech	69. I find it hard to communicate clearly what I want to say to people.	62	BAPQ	Pragmatic Language	14	People ask me to repeat things I’ve said because they don’t understand.	NA	NA	NA	NA	NA
SPQ	Odd Speech	7. People sometimes find it hard to understand what I am saying.	62	BAPQ	Pragmatic Language	14	People ask me to repeat things I’ve said because they don’t understand.	NA	NA	NA	NA	NA
SPQ	Constricted affect	17. I am not good at expressing my true feelings by the way I talk and look.	70	CATI	Communication	19	I can tell how people feel from their facial expressions	NA	NA	NA	NA	NA
SPQ	Constricted affect	35. My "nonverbal" communication (smiling and nodding during a conversation) is not very good.	70	CATI	Communication	19	I can tell how people feel from their facial expressions	NA	NA	NA	NA	NA
SPQ	Excessive social anxiety	71. I feel very uneasy talking to people I do not know well.	59	BFI	Sociability	43	I see myself as someone who is outgoing	9	AQ	Social skill	11	I find social situations easy
SPQ	Excessive social anxiety	38. Do you often feel	59	BFI	Sociability	43	I see myself as someone who	9	AQ	Social skill	11	I find social situations

		nervous when you are in a group of unfamiliar people?					is outgoing					easy
SPQ	No Closed Friends	6. I have little interest in getting to know other people.	66	BAPQ	Aloof	31	I prefer to be alone rather than with others.	NA	NA	NA	NA	NA
SPQ	Suspiciousness	59. I often feel that others have it in for me.	99	SPQ	Suspiciousness	18	Do you often feel that other people have it in for you?	NA	NA	NA	NA	NA
SPQ	Ideas of reference	1. Do you sometimes feel that things you see on the TV or read in the newspaper have a special meaning for you?	102	SPQ	Ideas of reference	28	Have you ever noticed a common event or object that seemed to be a special sign for you?	NA	NA	NA	NA	NA
SPQ	Ideas of reference	37. Do you sometimes see special meanings in advertisements, shop windows, or in the way things are arranged around you?	102	SPQ	Ideas of reference	28	Have you ever noticed a common event or object that seemed to be a special sign for you?	NA	NA	NA	NA	NA
SPQ	Odd Speech	16. I sometimes jump quickly from one topic to another when speaking.	105	SPQ	Odd Speech	34	I often ramble on too much when speaking.	NA	NA	NA	NA	NA
SPQ	Odd Speech	58. Do you tend to wander off the topic when having a conversation?	105	SPQ	Odd Speech	34	I often ramble on too much when speaking.	NA	NA	NA	NA	NA
SPQ	Odd Speech	72. People occasionally comment that my conversation is confusing.	105	SPQ	Odd Speech	34	I often ramble on too much when speaking.	NA	NA	NA	NA	NA

SPQ	Ideas of reference	63. Do you sometimes feel that people are talking about you?	110	SPQ	Ideas of reference	53	When you see people talk- ing to each other, do you often wonder If they are talking about you?	NA	NA	NA	NA	NA
SPQ	Odd Beliefs or Magical Thinking	21. Are you sometimes sure that other people can tell what you are thinking?	111	SPQ	Odd Beliefs or Magical Thinking	55	Have you ever felt that you are communicating with another person telepathically (by mind-reading)?	NA	NA	NA	NA	NA
SPQ	Odd Beliefs or Magical Thinking	12. Do you believe in telepathy (mInd-readIng)?	111	SPQ	Odd Beliefs or Magical Thinking	55	Have you ever felt that you are communicating with another person telepathically (by mind-reading)?	NA	NA	NA	NA	NA
SPQ	Unusual Perceptual Experiences	4. Have you often mistaken objects or shadows for people, or noises for voices?	106	SPQ	Unusual Perceptual Experiences	40	Have you ever seen things invisible to other people?	NA	NA	NA	NA	NA
WS	Social anhedonia	10. People are usually better off if they stay aloof from emotional involvements with most others.	54	BFI	Agreeableness	33	I see myself as someone who is sometimes cold and distant	NA	NA	NA	NA	NA
WS	Social anhedonia	5. I’m much too independent to really get involved with other people.	54	BFI	Agreeableness	33	I see myself as someone who is sometimes cold and distant	NA	NA	NA	NA	NA
WS	Social anhedonia	11. If given the choice, I would much rather be with others than be alone.	66	BAPQ	Aloof	31	I prefer to be alone rather than with others.	NA	NA	NA	NA	NA

WS	Social anhedonia	12. Although there are things that I enjoy doing by myself, I usually seem to have more fun when I do things with other people.	66	BAPQ	Aloof	31	I prefer to be alone rather than with others.	NA	NA	NA	NA	NA
WS	Social anhedonia	3. I prefer watching television to going out with other people.	66	BAPQ	Aloof	31	I prefer to be alone rather than with others.	NA	NA	NA	NA	NA
WS	Social anhedonia	1. Having close friends is not as important as many people say.	117	WS	Social anhedonia	4	Just being with friends can make me feel really good	NA	NA	NA	NA	NA
WS	Social anhedonia	13. I feel pleased and gratified as I learn more and more about the emotional life of my friends.	30	B2	Social Self-Regulation	2	I tend to be compassionate	NA	NA	NA	NA	NA
WS	Magical ideation	1. I have felt that there were messages for me in the way things were arranged, like in a store window.	102	SPQ	Ideas of reference	28	Have you ever noticed a common event or object that seemed to be a special sign for you?	NA	NA	NA	NA	NA
WS	Perceptual aberration	2. I have sometimes felt confused as to whether my body was really my own.	104	SPQ	Odd Beliefs or Magical Thinking	3	Have you had experiences with the supernatural?	NA	NA	NA	NA	NA
WS	Perceptual aberration	1. Occasionally it has seemed as if my body had taken on the appearance of another	104	SPQ	Odd Beliefs or Magical Thinking	3	Have you had experiences with the supernatural?	NA	NA	NA	NA	NA

		person's body.										
WS	Perceptual aberration	15. At times I have wondered if my body was really my own.	104	SPQ	Odd Beliefs or Magical Thinking	3	Have you had experiences with the supernatural?	NA	NA	NA	NA	NA
WS	Perceptual aberration	8. I have sometimes felt that some part of my body no longer belongs to me.	104	SPQ	Odd Beliefs or Magical Thinking	3	Have you had experiences with the supernatural?	NA	NA	NA	NA	NA
WS	Magical ideation	13. I think I could learn to read others' minds if I wanted to.	111	SPQ	Odd Beliefs or Magical Thinking	55	Have you ever felt that you are communicating with another person telepathically (by mind-reading)?	NA	NA	NA	NA	NA
WS	Magical ideation	6. I have sometimes felt that strangers were reading my mind.	111	SPQ	Odd Beliefs or Magical Thinking	55	Have you ever felt that you are communicating with another person telepathically (by mind-reading)?	NA	NA	NA	NA	NA
WS	Magical ideation	10. I have sometimes been fearful of stepping on sidewalk cracks.	115	WS	Magical ideation	5	At times I perform certain little rituals to ward off negative influences.	NA	NA	NA	NA	NA
WS	Perceptual aberration	4. Sometimes I have felt that I could not distinguish my body from other objects around me.	116	WS	Perceptual aberration	6	Sometimes I have had feelings that I am united with an object near me.	NA	NA	NA	NA	NA
WS	Perceptual aberration	5. I have felt that something outside my body was a part of my body.	116	WS	Perceptual aberration	6	Sometimes I have had feelings that I am united with an object near me.	NA	NA	NA	NA	NA
WS	Social anhedonia	7. I don't really	117	WS	Social anhedonia	4	Just being with	NA	NA	NA	NA	NA

		feel very close to my friends.					friends can make me feel really good					
WS	Social anhedonia	9. Knowing that I have friends who care about me gives me a sense of security.	117	WS	Social anhedonia	4	Just being with friends can make me feel really good	NA	NA	NA	NA	NA

SM11. Item-Level Reliability and Validity Analyses

Our study design prioritized feasibility in the field and participant burden: the questionnaire was deliberately fragmented into three different subsets, such that no participant completed the full set of items within any given construct. This design makes conventional measures of internal consistency (e.g., Cronbach's α, McDonald's ω) inapplicable, as these require complete within-person data across all items in a subscale. Instead, we employed item-level correlation approaches, which we consider more appropriate for this phase of adaptation. Specifically, we compared inter-item correlations within versus between scales (Spearman's ρ, Mann-Whitney test), computed an HTMT-like (HTMT: Heterotrait-Monotrait ratio of correlations, Henseler et al. 2015) ratio as an index of discriminant validity, and examined corrected item-total correlations relative to the strongest out-of-scale correlations (Wilcoxon signed-rank test). The HTMT-like ratio is the mean of the absolute correlations between items belonging to different scales (heterotrait) divided by the mean of the absolute correlations between items of the same scale (monotrait). It provides a summary index of discriminant validity, with lower values indicating that items correlate more strongly within their intended scale than across different scales. An HTMT value below 0.85 (sometimes 0.90 are typically considered evidence of adequate discriminant validity.

These analyses did not show strong evidence that items grouped more coherently within than between their intended subscales (median within-scale $\rho = 0.020$, IQR = (-0.130; 0.189) vs. between-scale $\rho = 0.031$, IQR = (-0.146; 0.204); Wilcoxon $p = 0.42$; HTMT-like = 1.07). Item-total correlations likewise failed to demonstrate a systematic within-scale advantage. In our data, the HTMT-like ratio was 10.7, indicating that average correlations between items from different scales were as high as, or even higher than, correlations among items within the same scale. This suggests limited evidence for discriminant validity of the intended subscales in the present sample. These outcomes reflect the limitations of the study design: fragmenting the questionnaire necessarily reduced the number of items per construct and the power to detect subscale-specific coherence.

Participants' responses by psychopathological dimension of the final instrument (n=117) of only the original items by scale (e.g., not counting redundancy)

Note: The results presented here should not be interpreted as sum scores or as indicative of prevalence by dimension. They are based on a limited sample size, and the original scales were substantially modified with respect to the number of items, linguistic formulation, and content.

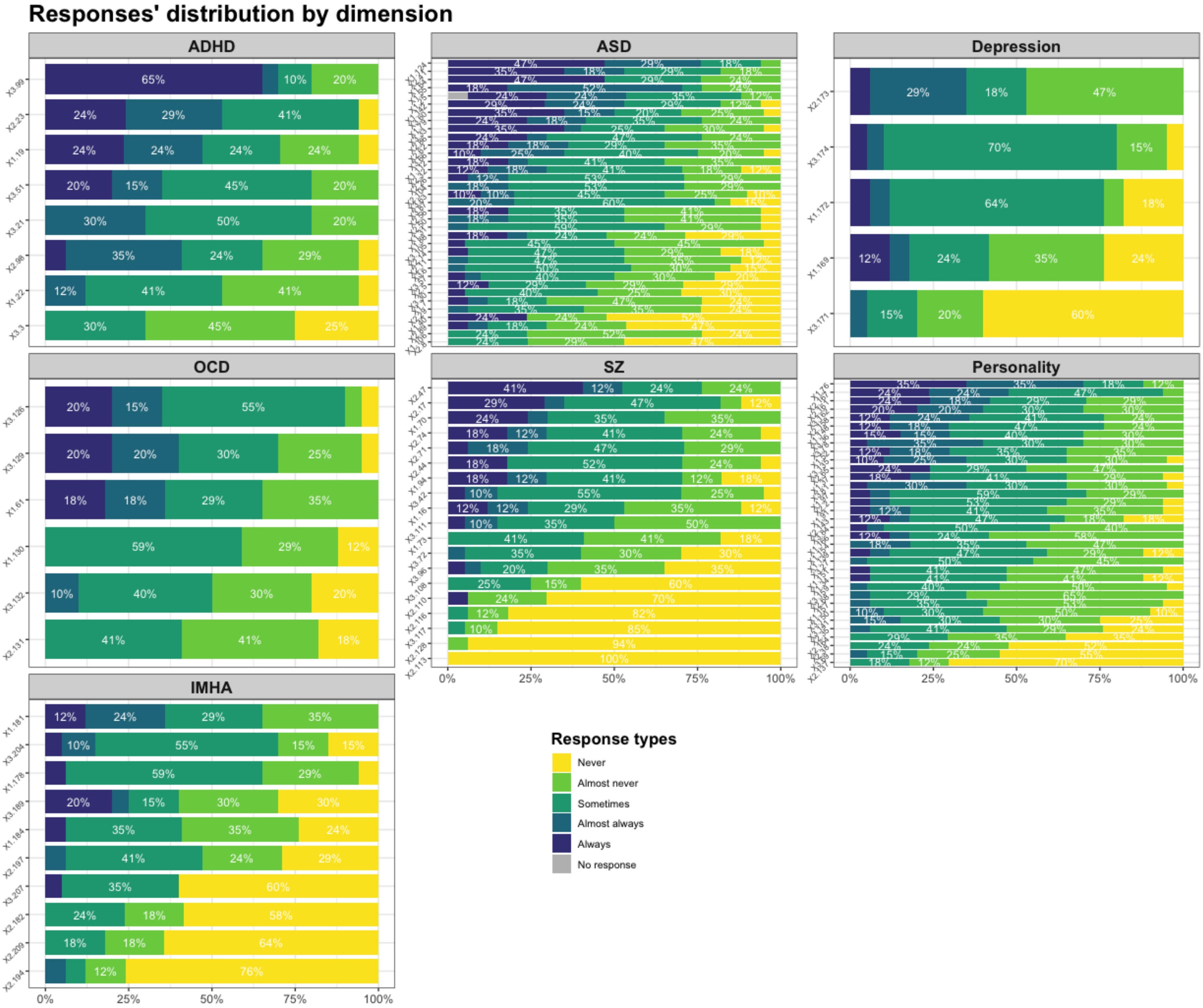

Responses' distribution by dimension
ADHD
ASD
Depression
OCD
SZ
Personality
IMHA
0%
25%
50%
75%
100%
Response types
Never
Almost never
Sometimes
Almost always
Always
No response
